# Adipose-derived mesenchymal stem cell exosomes ameliorate copper metabolism dysregulation and reduce cuproptosis caused by liver IRI

**DOI:** 10.3389/fvets.2026.1895340

**Published:** 2026-07-15

**Authors:** Lei Cao, Pujun Li, Yajun Ma, Xiangyu Lu, Yue Wang, Hongbin Wang, Jiantao Zhang, Tao Liu

**Affiliations:** 1 College of Veterinary Medicine, Northeast Agricultural University, Harbin, China; 2 Heilongjiang Provincial Key Laboratory of Pathogenic Mechanism for Animal Disease and Comparative Medicine, Harbin, China

**Keywords:** adipose-derived mesenchymal stem cell, copper metabolism, cuproptosis, exosome, hepatic ischemia reperfusion injury

## Abstract

**Introduction:**

Hepatic ischemia reperfusion injury is an important pathological factor leading to complications after hepatectomy and transplantation. Although cuproptosis has been reported as a new paradigm of programmed death triggered by copper homeostasis imbalance, its regulatory mechanisms and intervention strategies in liver IRI remain to be fully elucidated. The purpose of this study was to reveal the role of cuproptosis in liver IRI, and to elucidate the molecular mechanism by which adipose-derived stem cell exosomes (ADSC-Exos) exert therapeutic effects by regulating copper metabolism.

**Methods:**

Rat IRI models were established to evaluate copper metabolism dysregulation and cuproptosis activation. Subsequently, miniature pig models underwent laparoscopic IRI induction to assess ADSC-Exos’s effects on copper homeostasis restoration over 7 days post-injury.

**Results:**

We found that liver IRI disrupts copper homeostasis through a dual pathway: it inhibits membrane transporters CTR1 and ATP7B to affect copper ion excretion, and down-regulates intracellular copper chaperones ATOX1, CCS and COX17 expression, resulting in intracellular copper metabolism disorders. Excessive copper ions will bind to the lipoylated protein DLAT, induce its oligomerization and mitochondrial Fe-S cluster protein depletion, eventually leading to cuproptosis in hepatocytes and aggravating IRI. The intervention of ADSC-Exos can effectively regulate the disorder of copper metabolism in hepatocytes, inhibit the occurrence of cuproptosis, and reduce liver IRI.

**Discussion:**

This study first confirmed the damage mechanism of cuproptosis pathway caused by liver IRI, and revealed the regulatory mechanism of ADSC-Exos to hinder the process of cuproptosis by repairing the copper metabolism pathway of hepatocytes.

## Graphical abstract

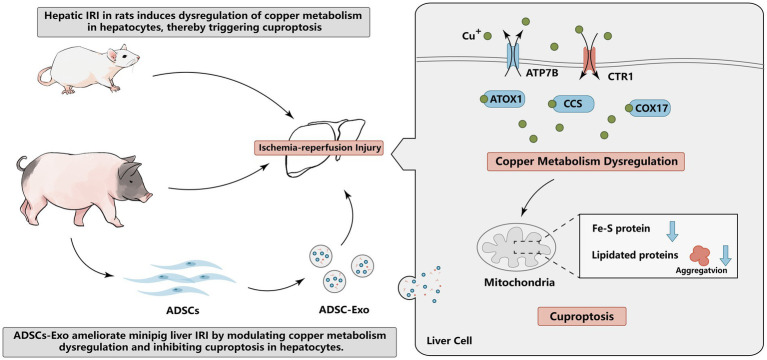

## 1 Introduction

In the process of liver transplantation and hepatectomy, it is inevitable to cause liver injury including hepatic ischemia reperfusion injury (IRI), which seriously affects the recovery of postoperative liver function and even induces liver failure (1). Therefore, in-depth analysis of the molecular mechanism of IRI and the establishment of effective prevention and treatment strategies have become the key scientific issues to be solved in the field of liver surgery. In the past, the mechanism of liver IRI was based on the molecular mechanism of cell death, such as autophagy, pyroptosis, ferroptosis and necroptosis; it has been well explained from the perspective of the role of various types of liver cells in ischemia–reperfusion injury and the resulting inflammatory response at the cytological level (2). It is worth noting that in 2022, Tsvetkov et al. found a new form of cell death caused by excessive copper in cells, namely cuproptosis, which provided a new perspective for the study of IRI mechanism (3). However, although bioinformatic analyses have revealed the importance of cuproptosis-related gene changes in hepatic IRI, the *in vivo* functional evidence and specific execution mechanisms of cuproptosis remain to be fully elucidated (4).

Cuproptosis is a copper-dependent cell death pathway mediated by protein lipoylation (5). Key mitochondrial enzyme complexes in the tricarboxylic acid (TCA) cycle, including pyruvate dehydrogenase and *α*-ketoglutarate dehydrogenase complexes, require lipoic acid as a cofactor for protein lipoylation to maintain physiological function (6). The binding of free copper ions to lipoylated proteins within mitochondria induces their abnormal oligomerization, coupled with depletion of Fe-S cluster proteins. This triggers acute mitochondrial proteotoxic stress, culminating in cuproptosis (3). Recent studies have pointed out that myocardial IRI can induce cuproptosis and aggravate myocardial cell injury by reducing the level of lipoyl synthase (LIAS) (7), suggesting that cuproptosis may play an important role in the process of IRI.

The liver serves as the primary copper reservoir in mammals, exhibiting the highest tissue copper ion concentration in the organism (8). As the central hub of systemic copper metabolism, the liver maintains unique homeostatic machinery: Dietary copper absorbed through the gastrointestinal tract is transported to hepatocytes, where it enters via copper transporter 1 (CTR1) (9). Intracellular copper is either stored through metallothionein complexation or delivered by chaperones to cuproenzymes requiring copper as an essential cofactor for catalytic functions (10). The excessive copper ions in hepatocytes are mainly excreted into bile through ATPase copper transporting beta (ATP7B), which is excreted through the bile duct (11). Imbalance in this precisely controlled system, resulting from either excessive copper intake or impaired excretion, leads to intracellular copper accumulation and subsequent cuproptosis (12).

Adipose-derived mesenchymal stem cells (ADSCs) are a type of mesenchymal stem cells (MSCs) isolated from adipose tissue. Due to the widespread distribution of adipose tissue and the minimally invasive nature of its collection, ADSCs have become an MSC source characterized by abundant availability, easy accessibility, and low ethical concerns (13). The exosomes secreted by ADSCs contain various biological information from these cells (14, 15). They have been shown to not only reduce risks of cell transplantation but also mimic the therapeutic effects of the parent cells (16). Previously, we have demonstrated that ADSCs-derived exosomes (ADSC-Exos) can alleviate hepatic IRI by regulating mitochondrial quality control dysfunction (17) and cellular metabolic disorders in hepatocytes (18). Here, we further propose that hepatic IRI suppresses copper metabolism in hepatocytes, triggering excessive copper ion accumulation and subsequent cuproptosis. ADSC-Exos can ameliorate hepatic IRI by regulating copper metabolism and cuproptosis in hepatocytes.

## 2 Materials and methods

### 2.1 Liver IRI rat models

Twelve male Sprague–Dawley rats, aged 6–8 weeks and weighing 220–250 g, were purchased from Liaoning Changsheng Biotechnology Co., Ltd. (Shenyang, Liaoning, China). All experiments were approved by the Animal Ethics Committee of Northeast Agricultural University. They were provided with adequate water and food and then randomly divided into a sham group and an IRI group, with 6 rats in each group. The rat liver IRI model was constructed using the previous method (16). The rats were anesthetized with isoflurane (RWD, Shenzhen, China) and then operated. After exposing the liver, Glisson ‘s blood supply to the middle and right lobes of the liver was blocked with a non-invasive vascular clamp to perform liver ischemia. The abdominal cavity was temporarily closed during the ischemic period to maintain the temperature of the intra-abdominal organs. After 15 min of ischemia, the left hepatic lobe was resected. Following 30 min of total ischemia, the vascular clamp was removed to initiate reperfusion. The abdominal cavity was thoroughly irrigated and then closed. After 24 h of operation, the rats were sacrificed by cervical dislocation, and blood and liver tissue samples were collected for analysis. The sham group underwent liver lobe mobilization only, followed by abdominal closure, without inducing hepatic IRI.

### 2.2 Liver IRI miniature pig models

Eighteen healthy 4-5-month-old miniature pigs were randomly divided into sham group, IRI group and Exo group, with 6 pigs in each group. All experiments were approved by the Animal Ethics Committee of Northeast Agricultural University (Bioethics protocol number: NEAUEC2024 03 146). Laparoscopic minimally invasive surgery was used in the same way to construct a liver IRI model on miniature pigs, and the same drug concentration and dose were used to intervene in the injury (17). Briefly, minipigs underwent respiratory anesthesia and were positioned in dorsal recumbency. A four-port laparoscopic surgical approach was established. The right hepatic lobe was subjected to ischemia for 60 min to construct liver IRI, and then the left hepatic lobe was removed. Immediately after model establishment, the therapeutic agents were administered via portal vein injection: 5 mL of PBS for the IRI group, and 5 mL of PBS containing 200 μg of exosomes for the Exo group. All pigs survived after the experiment was completed. All the animal experiments were conducted according to ARRIVE guidelines 2.0 (Animal Research: Reporting of *In Vivo* Experiments). The experimental samples obtained from each group were assigned numbers, and three samples were then randomly selected using a random number generator for further laboratory testing. It is necessary to add that the samples selected for each experiment are not the same.

### 2.3 Acquisition of ADSCs and ADSC-Exos

ADSCs and ADSC-Exos were isolated using a previous method (19). Briefly, adipose tissue was surgically harvested from the inguinal region of minipigs. The tissue was then digested with collagenase type I to isolate ADSCs. The cells were cultured in low-glucose DMEM (HyClone, Logan, USA) supplemented with 10% fetal bovine serum (FBS, HyClone, Logan, USA), 1% L-glutamine, and 1% penicillin/streptomycin, under standard conditions (37 °C, 5% CO₂, and 95% air). ADSCs were then expanded to passages 3–4 for exosome isolation. When the cells reached approximately 90% confluence, the medium was replaced with serum-free medium for a 24-h starvation period. Following this, the supernatant was collected, and exosomes were isolated and purified using ultrafiltration and ultracentrifugation.

### 2.4 Histological analysis

Fresh liver tissue samples were fixed in 4% paraformaldehyde for 24 h. After paraffin embedding, sectioning, hematoxylin and eosin (H&E) staining, the morphological changes of liver tissue were observed under a light microscope.

### 2.5 Blood biochemistry analysis

Aspartate Aminotransferase (AST), Alanine Aminotransferase (ALT), Alkaline Phosphatase (ALP) and Total Bilirubin (TBIL) level in the serum was measured by a blood biochemistry analyzer (TBA-2000FR, Japan).

### 2.6 Measurement of Cu^2+^ content

The same amount of liver tissue was ground into 10% homogenate, and the supernatant was taken for detection after centrifugation. According to the instructions provided in the Cu^2+^ colorimetric test kit (Elabscience, E-BC-K300-M, Wuhan, China), the OD value of the sample was measured at 580 nm using a microplate reader.

### 2.7 Real-time quantitative PCR

Total RNA was extracted from liver tissue by Trizol method, reverse transcribed into cDNA (Vazyme, Wuhan, China) and labeled with SYBR Green I fluorescent dye (Innovation, Beijing, China). Subsequently, the target gene was amplified using a two-step method in LightCycler 480 (Roche, Indianapolis, USA). The relative gene expression was calculated by 2^-ΔΔCt^ method with *β*-actin as the internal reference gene. All primers were custom-synthesized by BGI (China). The primer sequences are listed in Supplementary Tables 1, 2.

### 2.8 Western blotting

Total protein was extracted from liver tissue using RIPA lysis buffer. The extracted proteins were separated by SDS-PAGE using gels (Epizyme, Shanghai, China), followed by electroblotting transfer onto PVDF membranes. After blocking with rapid blocking buffer, membranes were sequentially incubated with primary antibodies overnight at 4 °C, then with secondary antibodies for 2 h (room temperature). Blots were washed with TBST between each incubation step. Protein bands were detected using enhanced ECL reagent (Meilunbo, Dalian, China) on a Tanon 5,200 system (Tanon, Shanghai, China). Mean optical density values were quantified with ImageJ software. Detailed antibody information is provided in Table 1.

**Table 1:** Antibody related information.

Name	Species	Supplier	Cat no.
anti-FDX1	Pig	Abmart (Shanghai, China)	TD7950
anti-FDX1	Rat	Abclonal (Woburn, USA)	A20895
anti-LIAS	Rat/Pig	Proteintech (Wuhan, China)	44577-1-AP
anti-ACO2	Rat/Pig	Proteintech (Wuhan, China)	67509-1-lg
anti-SDHB	Rat/Pig	Proteintech (Wuhan, China)	10620-1-AP
anti-HSP70	Rat/Pig	Proteintech (Wuhan, China)	10995-1-AP
anti-CTR1	Rat/Pig	Proteintech (Wuhan, China)	27499-1-AP
anti-ATP7B	Rat/Pig	Bioss (Beijing, China)	Bs-1718R
anti-DLST	Rat/Pig	Abmart (Shanghai, China)	TD13671
anti-DLAT	Pig	Abmart (Shanghai, China)	PK69694
anti-DLAT	Rat	Abmart (Shanghai, China)	T58125
anti-Lipoic acid	Rat/Pig	Merck (Rahway, USA)	sc-101354
anti-actin	Rat/Pig	Proteintech (Wuhan, China)	66009-1-lg
anti-GADPH	Rat/Pig	Proteintech (Wuhan, China)	60004-1-lg
anti-β-tubulin	Rat/Pig	Abclonal (Woburn, USA)	A12289

### 2.9 Immunohistochemistry

Formalin-fixed tissues were embedded in paraffin and sectioned. Tissues sections (4 μm) were immunostained using the MaxVision Kit (Maixin Biol, KIT-5020) according to the manufacturer’s instructions. The primary antibodies were anit-ATP7B at 1: 100 dilution and anit-FDX1 at 1: 100 dilution. In all, 50 μL MaxVision reagent was applied to each slide. Color was developed with 0.05% diaminobenzidine and 0.03% H_2_O_2_ in 50 mM Tris–HCl (pH 7.6), and the slides were counterstained with hematoxylin. Finally, the slides were scanned using a digital pathological section scanner (Aperio CS2).

### 2.10 Immunofluorescence

Paraffin sections were dewaxed and rehydrated. Antigen recovery was accomplished by high-pressure heat recovery. Sections were then blocked with bovine serum albumin (BSA) for 30 min at room temperature, followed by incubation with primary antibody in PBS (anti-DLAT at 1:100 dilution) at 4 °C overnight. PBS After washing 3 times, and the nuclei were counterstained with DAPI and quenched for autofluorescence using an autofluorescence quencher (Servicebio, Beijing, China). Subsequently, the sections were sealed with an antifluorescence quencher (Servicebio, Beijing, China). Subsequently, the sections were observed by fluorescence microscopy.

### 2.11 Statistical analysis

Statistical analysis was performed using SPSS 25.0. Charting was performed using GraphPad Prism 8.0.2 soft-ware. Firstly, Shapiro–Wilk method is used to test the normality of data, and Brown-Forsythe method is used to test the homogeneity of variance. Once it meets the standards, a one-way ANOVA was used to analyze the differences be-tween groups. After obtaining the results, we used Tukey HSD for *post hoc* testing to determine the final significant differences. A *p*-value of <0.01 indicates a highly significant statistical difference, 0.01 < *p* < 0.05 indicates a significant statistical difference, and *p* > 0.05 indicates no significant statistical difference. Experimental data are presented as mean ± standard deviation (mean ± SD).

## 3 Results

### 3.1 Hepatic IRI induces copper metabolism disorder in rat hepatocytes and causes an increase in copper ion concentration

To observe the effects of IRI on hepatic tissue, liver tissues and serum were collected from rats at 24 h post-IRI for morphological observation and biochemical detection. As shown in Supplementary Figure S1A, compared with the sham group without modeling, IRI induced extensive pathological changes in hepatocytes, including vacuolar degeneration, cellular swelling, karyolysis, disorganized hepatic cord architecture, as well as focal necrosis and inflammatory cell infiltration. Furthermore, hepatic IRI caused markedly elevated serum levels of AST, ALT, ALP and TBIL (Supplementary Figures S1B–E), indicating severe impairment of both hepatic morphology and function.

**Figure 1:**
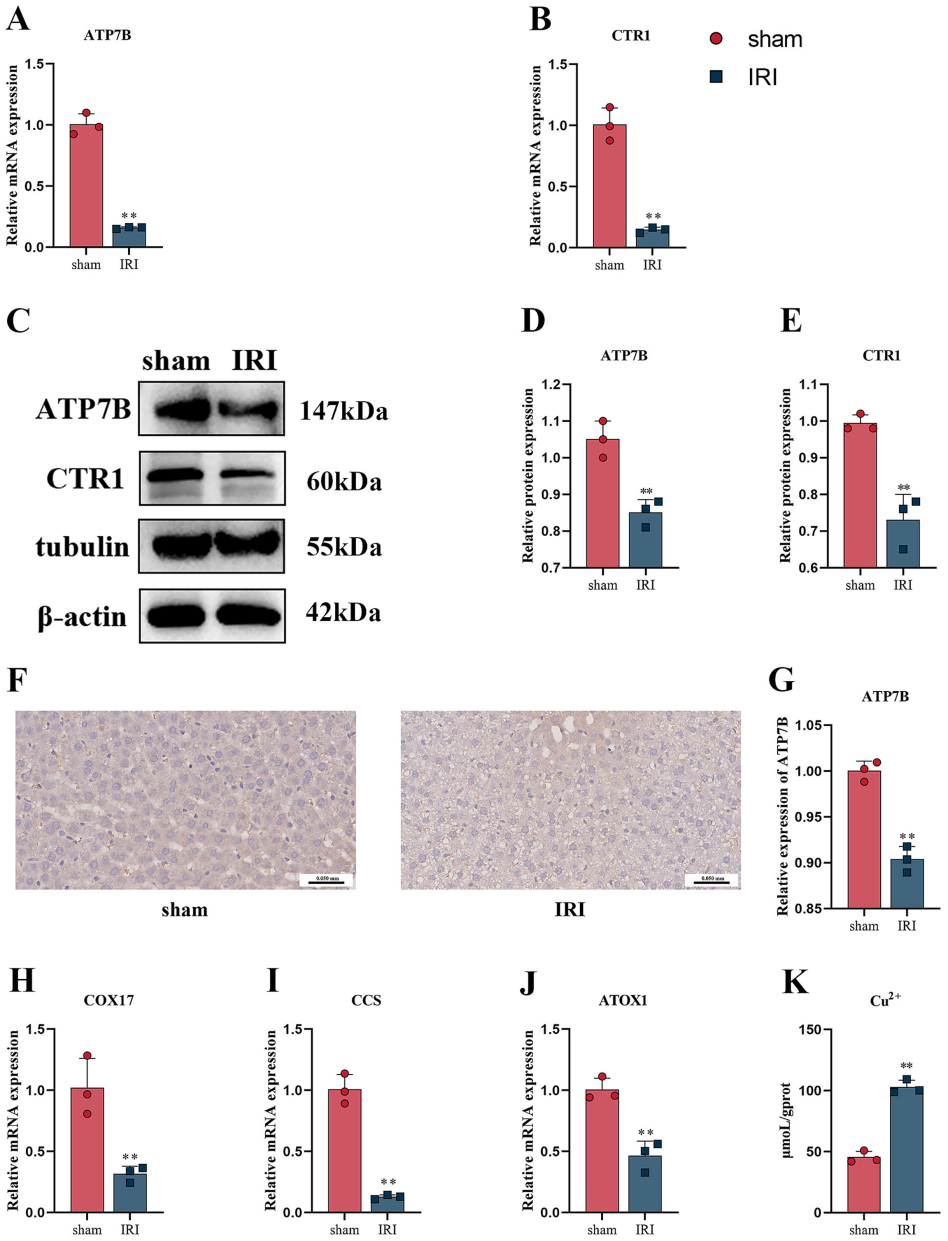
Liver IRI induced disorder of copper metabolism in rat hepatocytes and increased copper ion concentration in liver tissue. **(A,B)** qRT-PCR-assisted detection of mRNA coding for ATP7B and CTR1. **(C–E)** Western blot results and quantitative analysis of ATP7B and CTR1 in each group. **(F,G)** Immunohistochemical staining results and quantitative analysis of ATP7B content in liver tissue. **(H–J)** qRT-PCR-assisted detection of mRNA coding for COX17, CCS and ATOX1. **(K)** Results of Cu^2+^ content assay in liver tissues. Results are presented as mean ± SD (*n* = 3) * *p* < 0.05, * * *p* < 0.01 versus the Sham group, ^#^ *p* < 0.05, ^##^ *p* < 0.01 versus the IRI group.

To investigate the impact of hepatic IRI on copper metabolism in hepatocytes, we analyzed alterations in copper metabolism-related indicators in rat liver tissues post-IRI. Following hepatic IRI, both gene and protein expression levels of ATP7B and CTR1 in hepatocytes were markedly decreased (Figures 1A–E). Immunohistochemical analysis revealed markedly reduced and unevenly distributed ATP7B expression in hepatocytes, which was associated with disorganization of the hepatic cords (Figures 1F,G). Additionally, hepatic IRI disrupted intracellular copper transport mediated by copper chaperones (10), with significantly suppressed gene expression of cytochrome c oxidase 17 (COX17), copper chaperone for superoxide dismutase (CCS), and antioxidant 1 copper chaperone (ATOX1) post-injury (Figures 1H–J). Quantitative analysis demonstrated a pronounced elevation of Cu^2+^ content in liver tissues after IRI (Figure 1K). These findings collectively indicate that IRI induces dysregulation of copper ion transport in hepatocytes, ultimately leading to intracellular copper accumulation.

### 3.2 Hepatic IRI induces a reduction in Fe-S cluster proteins and an elevation in HSP70 expression within rat hepatocytes

**Figure 2:**
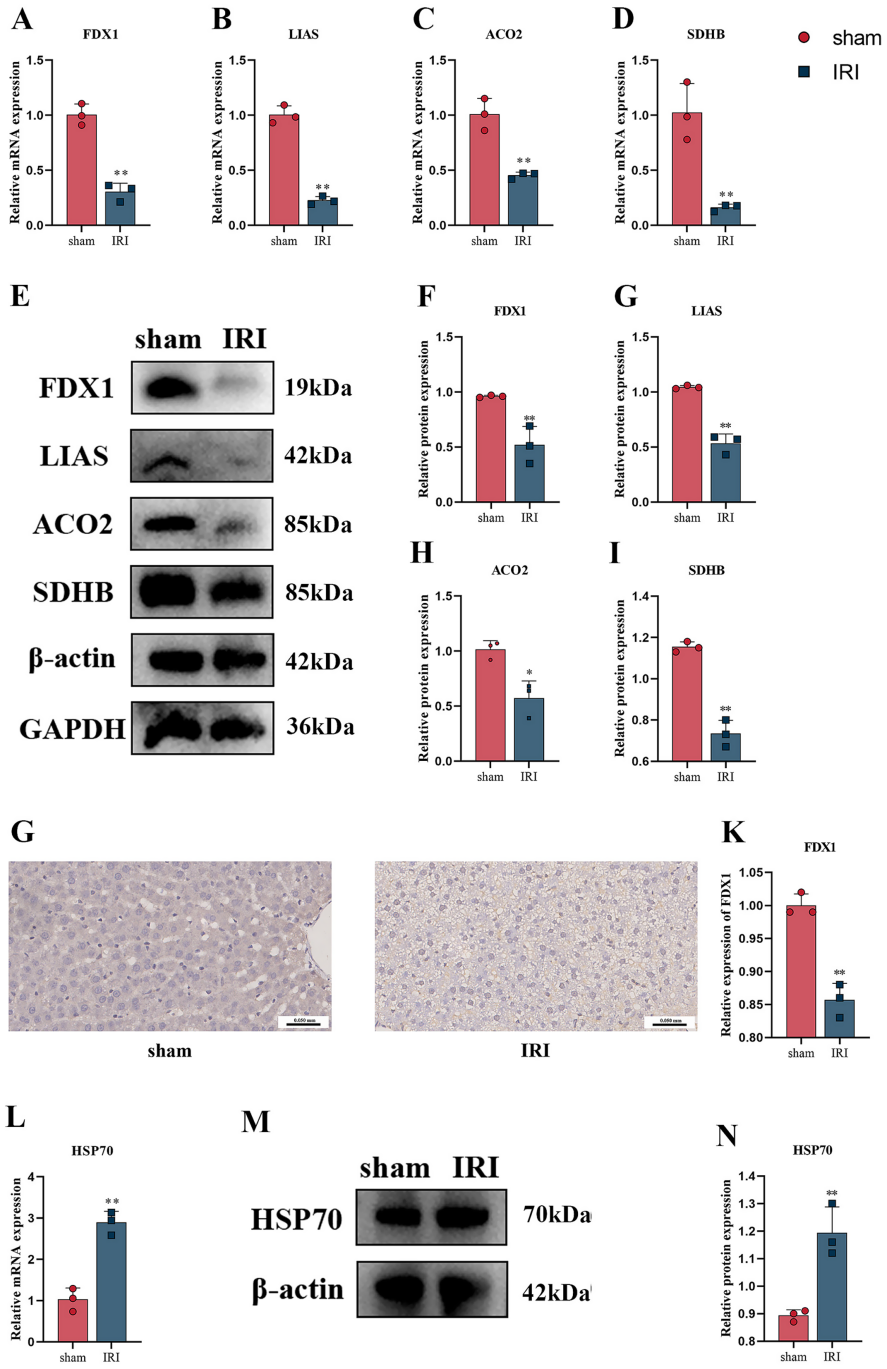
Liver IRI leads to the decrease of Fe-S cluster protein and the increase of HSP70 expression in rat hepatocytes. **(A–D)** qRT-PCR-assisted detection of mRNA coding for FDX1, LIAS, ACO2 and SDHB. **(E–I)** Western blot results and quantitative analysis of FDX1, LIAS, ACO2 and SDHB in each group. **(J,K)** Immunohistochemical staining results and quantitative analysis of FDX1 content in liver tissue. **(L)** qRT-PCR-assisted detection of mRNA coding for HSP70. **(M,N)** Western blot results and quantitative analysis of HSP70 in each group. Results are presented as mean ± SD (*n* = 3) * *p* < 0.05, * * *p* < 0.01 versus the Sham group, ^#^ *p* < 0.05, ^##^ *p* < 0.01 versus the IRI group.

To determine whether excessive copper ions in hepatocytes can trigger cuproptosis, we first examined the expression of Fe-S cluster proteins in liver tissues. qRT-PCR and Western blot analyses revealed that hepatic IRI significantly reduced both gene and protein expression levels of ferredoxin 1 (FDX1), LIAS, aconitase 2 (ACO2), and succinate dehydrogenase complex iron–sulfur subunit B (SDHB) (Figures 2A–I). The immunohistochemistry results showed that the expression of FDX1 in liver tissue decreased concurrently (20), and its distribution became abnormal, which was closely associated with the dissolution of hepatocyte nuclei (Figures 2J,K). Additionally, hepatic IRI caused a marked upregulation in both gene and protein expression of heat shock protein 70 (HSP70) (Figures 2L–N), suggesting that IRI triggers cellular stress responses (21). This further suggests that hepatic IRI may trigger cuproptosis (22).

### 3.3 Hepatic IRI induces a reduction in lipoylated proteins accompanied by their abnormal aggregation

**Figure 3:**
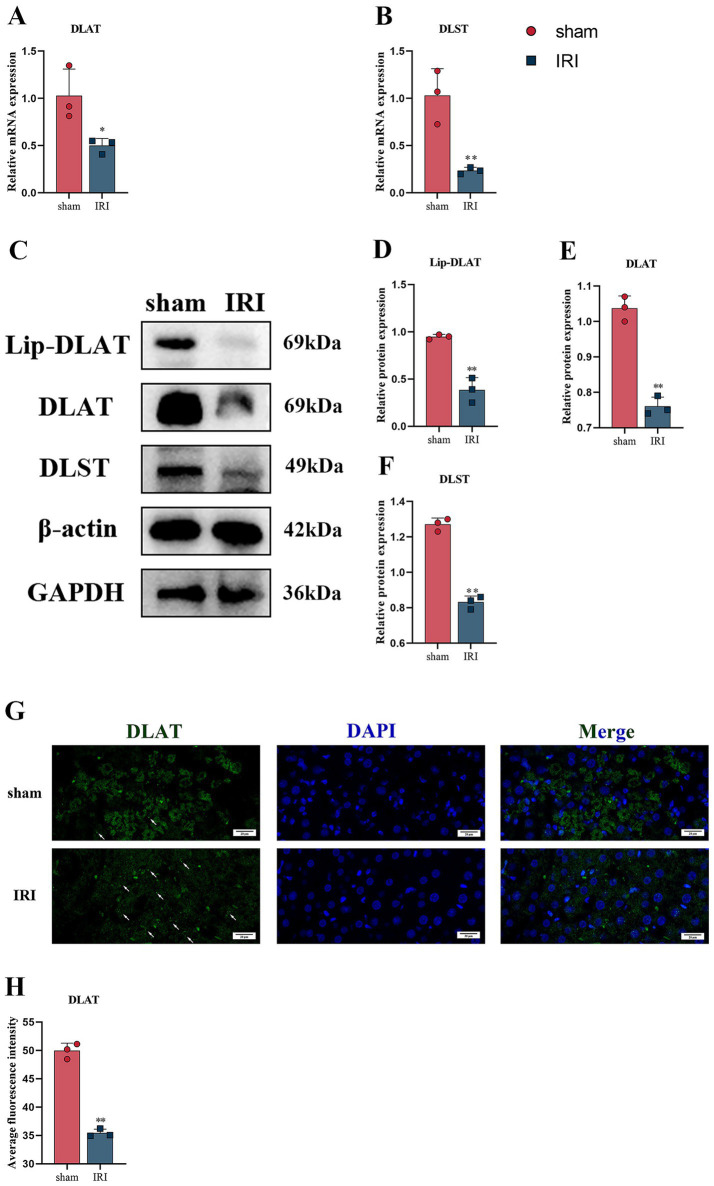
Liver IRI leads to the decrease of lipoylated protein and abnormal aggregation in rats. **(A,B)** qRT-PCR-assisted detection of mRNA coding for DLAT and DLST. **(C–F)** Western blot results and quantitative analysis of Lip-DLAT, DLAT and DLST in each group. **(G,H)** Immunofluorescence staining results, quantitative analysis of DLAT content in liver tissue. The areas where DLAT oligomerization occurs are indicated by white arrows. Results are presented as mean ± SD (*n* = 3) * *p* < 0.05, * * *p* < 0.01 versus the Sham group, ^#^ *p* < 0.05, ^##^ *p* < 0.01 versus the IRI group.

Another hallmark event of cuproptosis is the reduction of lipoylated proteins involved in the TCA cycle, such as dihydrolipoamide S-acetyltransferase (DLAT) and dihydrolipoamide S-succinyltransferase (DLST), which aggregate upon binding to copper ions (23). To investigate this, we first analyzed the expression of TCA cycle-associated lipoylated proteins. As shown in Figures 3A–F, liver IRI significantly reduced the gene and protein expression levels of DLAT and DLST in liver tissue. Furthermore, we also detected that it inhibited the lipoylation level of DLAT (Lip-DLAT). Immunofluorescence staining of DLAT revealed its subcellular distribution in hepatocytes (Figures 3G,H). Notably, although total DLAT fluorescence intensity markedly decreased post-IRI, prominent high-intensity punctate foci were observed, indicative of pathological protein aggregation (24). Collectively, these results demonstrate that hepatic IRI induces dysregulation of copper metabolism in hepatocytes, leading to excessive copper accumulation due to impaired efflux. This copper overload ultimately triggers cuproptosis.

### 3.4 ADSC-Exos effectively regulate the decrease in Fe-S cluster proteins caused by hepatic IRI in miniature pigs and reduce cellular stress

To evaluate the long-term regulatory effects of ADSC-Exos on hepatic IRI induced copper metabolism disorder and leveraging the high homology between miniature pigs and humans (25), we established a hepatic IRI model in miniature pigs using laparoscopic techniques. Liver tissues and serum samples were collected at postoperative days 1, 3, and 7 for copper metabolism-related analyses. HE staining of liver tissues (Supplementary Figure S2A) demonstrated that compared to the sham group without injury, the IRI group at day 1 post-surgery exhibited extensive vacuolar degeneration of hepatocytes, disorganized hepatic cord architecture, and pronounced inflammatory cell infiltration. By day 3, vacuolar degeneration was reduced, but hepatocyte swelling persisted, with normal histology restored only by day 7. In contrast, ADSC-Exos intervention significantly attenuated vacuolar degeneration in the IRI group as early as day 1, restored hepatocyte morphology to normal by day 3, and limited inflammatory cell infiltration predominantly to hepatic sinusoids. Moreover, ADSC-Exos intervention was shown to effectively reduce serum levels of AST, ALT, TBIL, and ALP at 1 d, and even at 3 d, after hepatic IRI (Supplementary Figures SB–E). These findings indicate that ADSC-Exos effectively mitigate IRI-induced hepatic structural injury and maintain normal liver function.

**Figure 4:**
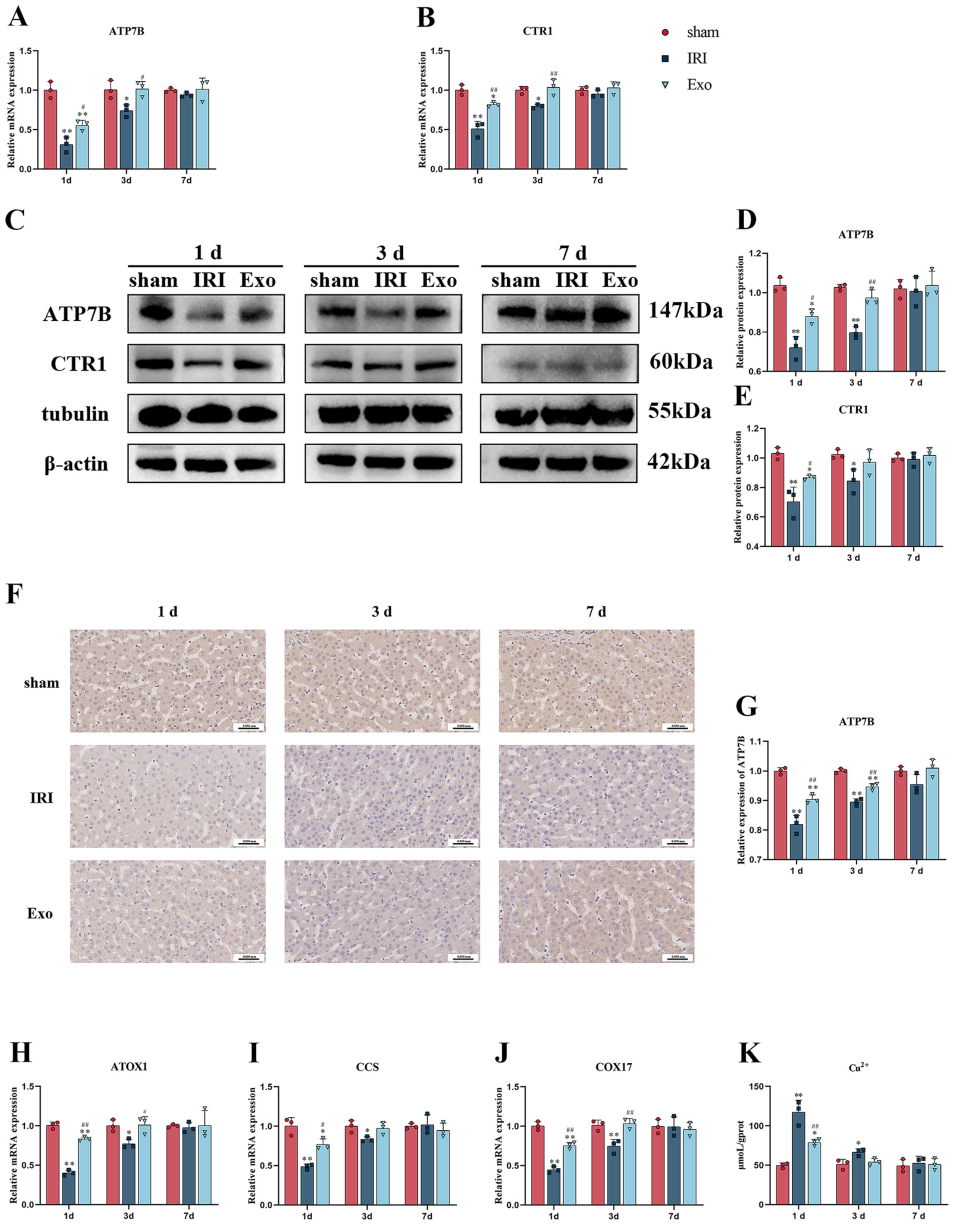
ADSC-Exos can effectively regulate the disorder of hepatocyte copper metabolism caused by liver IRI in miniature pigs and reduce the level of copper in tissues. **(A,B)** qRT-PCR-assisted detection of mRNA coding for ATP7B and CTR1. **(C–E)** Western blot results and quantitative analysis of ATP7B and CTR1 in each group. **(F,G)** Immunohistochemical staining results and quantitative analysis of ATP7B content in liver tissue. **(H–J)** qRT-PCR-assisted detection of mRNA coding for COX17, CCS and ATOX1. **(K)** Results of Cu^2+^ content assay in liver tissues. Results are presented as mean ± SD (*n* = 3) * *p* < 0.05, * * *p* < 0.01 versus the Sham group, ^#^ *p* < 0.05, ^##^ *p* < 0.01 versus the IRI group.

We next examined the effects of Exo on hepatocyte copper metabolism. qRT-PCR and Western blot analyses (Figures 4A–E) demonstrated that Exo effectively upregulated the expression of CTR1 and ATP7B in hepatocytes, restoring both proteins to normal levels by postoperative day 3. Immunohistochemical results (Figures 4F,G) showed that in miniature pigs, hepatic IRI injury significantly reduced ATP7B expression in hepatocytes as early as postoperative day 1. However, Exo treatment maintained ATP7B membrane localization and accelerated its recovery by days 1 and 3. Furthermore, Exo exhibited pronounced therapeutic effects on copper chaperones involved in intracellular copper transport (Figures 4H–J). The expression levels of ATOX1, CCS, and COX17 returned to baseline by day 3 with Exo intervention. Critically, measurements of hepatic copper ion concentrations confirmed Exo’s regulatory role in copper metabolism, effectively mitigating pathological copper accumulation (Figure 4K). Copper ion levels normalized by day 3 in the Exo group.

### 3.5 ADSC-Exos effectively regulate the decrease in Fe-S cluster proteins caused by hepatic IRI in miniature pigs and reduce cellular stress

**Figure 5:**
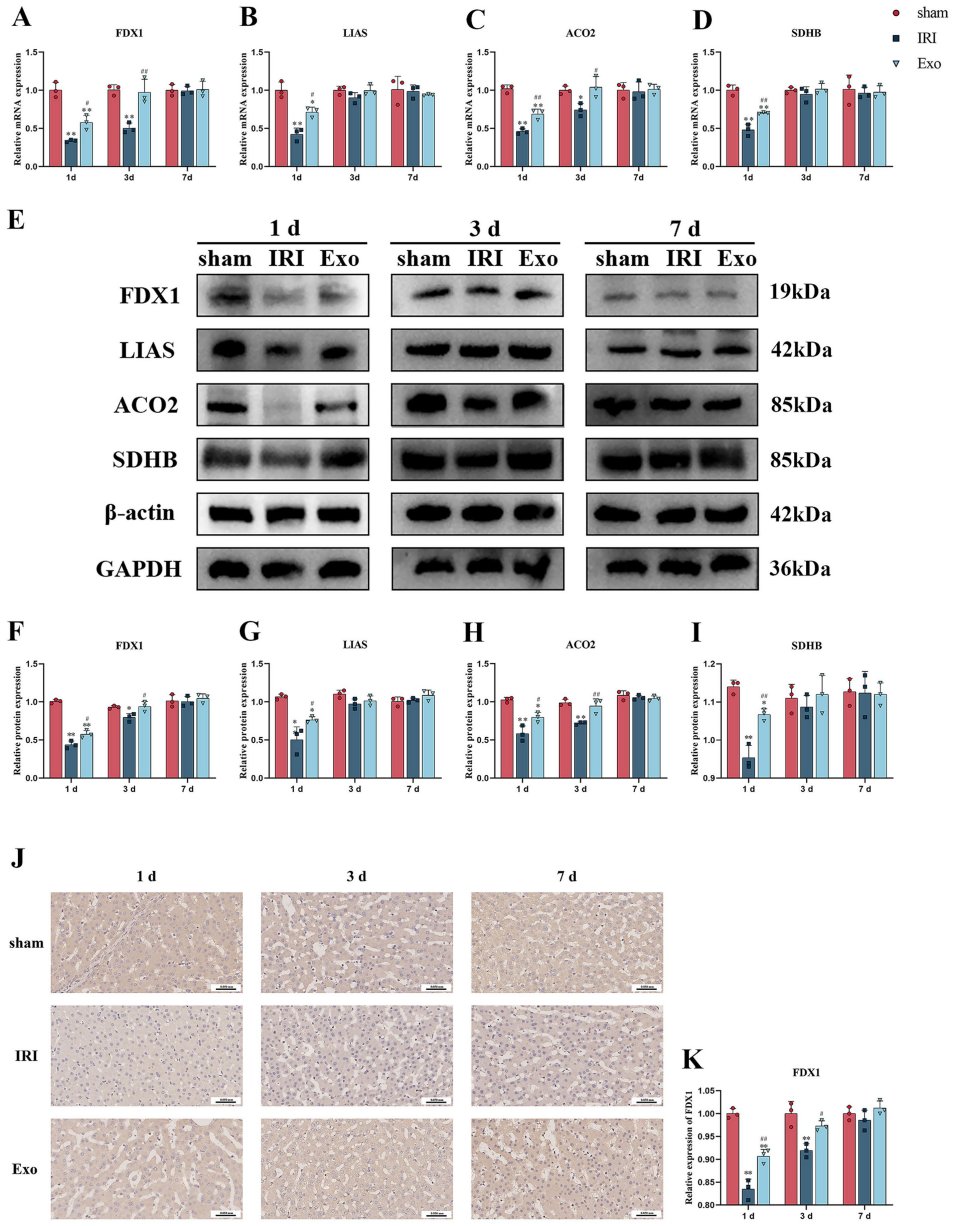
ADSCs-derived Exo effectively regulates the reduction of Fe-S cluster proteins caused by liver IRI in miniature pigs. **(A–D)** qRT-PCR-assisted detection of mRNA coding for FDX1, LIAS, ACO2 and SDHB. **(E–I)** Western blot results and quantitative analysis of FDX1, LIAS, ACO2 and SDHB in each group. **(J,K)** Immunohistochemical staining results and quantitative analysis of FDX1 content in liver tissue. Results are presented as mean ± SD (*n* = 3) * *p* < 0.05, * * *p* < 0.01 versus the Sham group, ^#^ *p* < 0.05, ^##^ *p* < 0.01 versus the IRI group.

**Figure 6:**
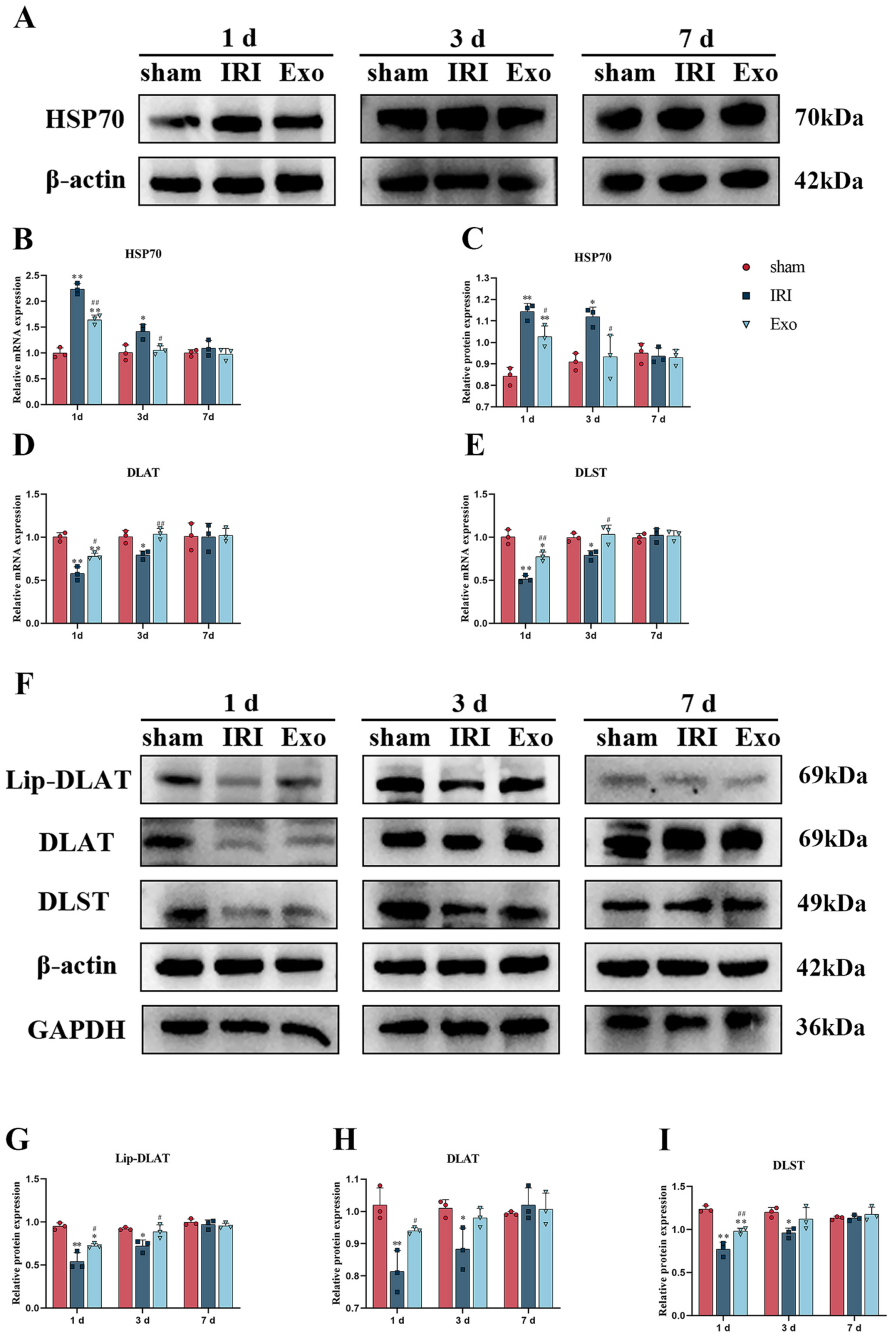
ADSC-Exos inhibits the expression of HSP70 and effectively regulates the reduction of lipoylated protein caused by liver IRI in miniature pigs. **(A–C)** The expression of HSP70 in liver tissue was detected by qRT-PCR and Western blot. **(D,E)** qRT-PCR-assisted detection of mRNA coding for DLAT and DLST. **(F–I)** Western blot results and quantitative analysis of Lip-DLAT, DLAT and DLST in each group. Results are presented as mean ± SD (*n* = 3) * *p* < 0.05, * * *p* < 0.01 versus the Sham group, ^#^ *p* < 0.05, ^##^ *p* < 0.01 versus the IRI group.

To investigate the impact of ADSC-Exos on cuproptosis, we first analyzed changes in the expression of Fe-S cluster proteins in liver tissues of miniature pigs. As shown in Figures 5A–I, ADSC-Exos effectively enhanced the expression of multiple Fe-S cluster proteins in post-IRI liver tissues and restored their levels to baseline by postoperative day 3. Immunohistochemical analysis of FDX1 (Figures 5J,K) demonstrated that its expression in liver tissue decreased following liver IRI, while exosome intervention preserved the normal expression level of this protein in liver tissue. The expression of HSP70 in liver tissue showed that (Figures 6A–C), after 3 days of IRI, the liver cells were still in the state of cell stress, and did not return to normal level until 7 days. Remarkably, ADSC-Exos not only mitigated hepatocyte stress but also expedited recovery from the stressed state within 3 days post-injury.

### 3.6 ADSC-Exos effectively alleviate the reduction of lipoylated proteins caused by hepatic IRI in miniature pigs and prevent their pathological aggregation

**Figure 7:**
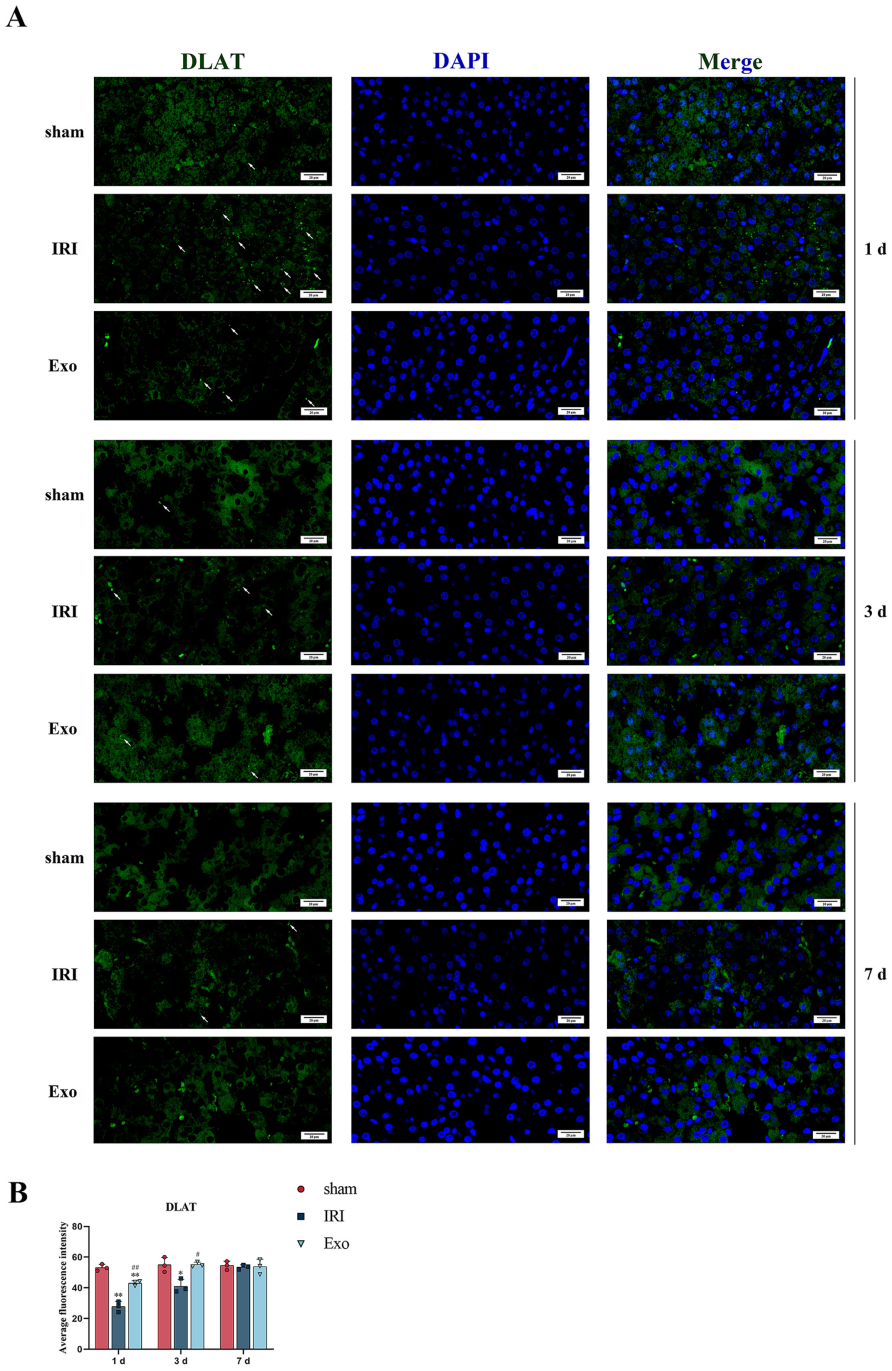
ADSC-Exos reduces abnormal DLAT aggregation caused by liver IRI. **(A-B)** Immunofluorescence staining results, quantitative analysis of DLAT content in liver tissue. The areas where DLAT oligomerization occurs are indicated by white arrows. Results are presented as mean ± SD (*n* = 3) *p < 0.05, **p < 0.01 versus the Sham group, #*p* < 0.05, ##*p* < 0.01 versus the IRI group.

To further explore the effects of Exo on another hallmark event of cuproptosis, we confirmed their regulatory role in lipoylated proteins. First, Exo effectively increased the expression of DLAT and DLST, two proteins involved in the TCA cycle, in hepatocytes (Figures 6D–F,H,I) and restored their levels to normal within 3 days after IRI. Additionally, Exo intervention maintained lipoylation modification levels of DLAT (Figures 6F,G), achieving recovery by day 3 post IRI. Immunofluorescence analysis of DLAT (Figure 7) showed that Exo significantly reduced abnormal aggregation at day 1 after IRI. By day 3, compared to the severe aggregation observed in the IRI group, no pathological aggregation was detected in the Exo treated group.

The comprehensive results demonstrate that Exo intervention can effectively alleviate copper metabolism disorders caused by IRI, thereby stabilizing the expression of Fe-S cluster proteins in liver tissue, ensuring normal expression and lipoylation of proteins involved in the TCA cycle, preventing abnormal aggregation triggered by the binding of copper ions to lipoylated proteins, and ultimately inhibiting cuproptosis.

## 4 Discussion

**Figure 8:**
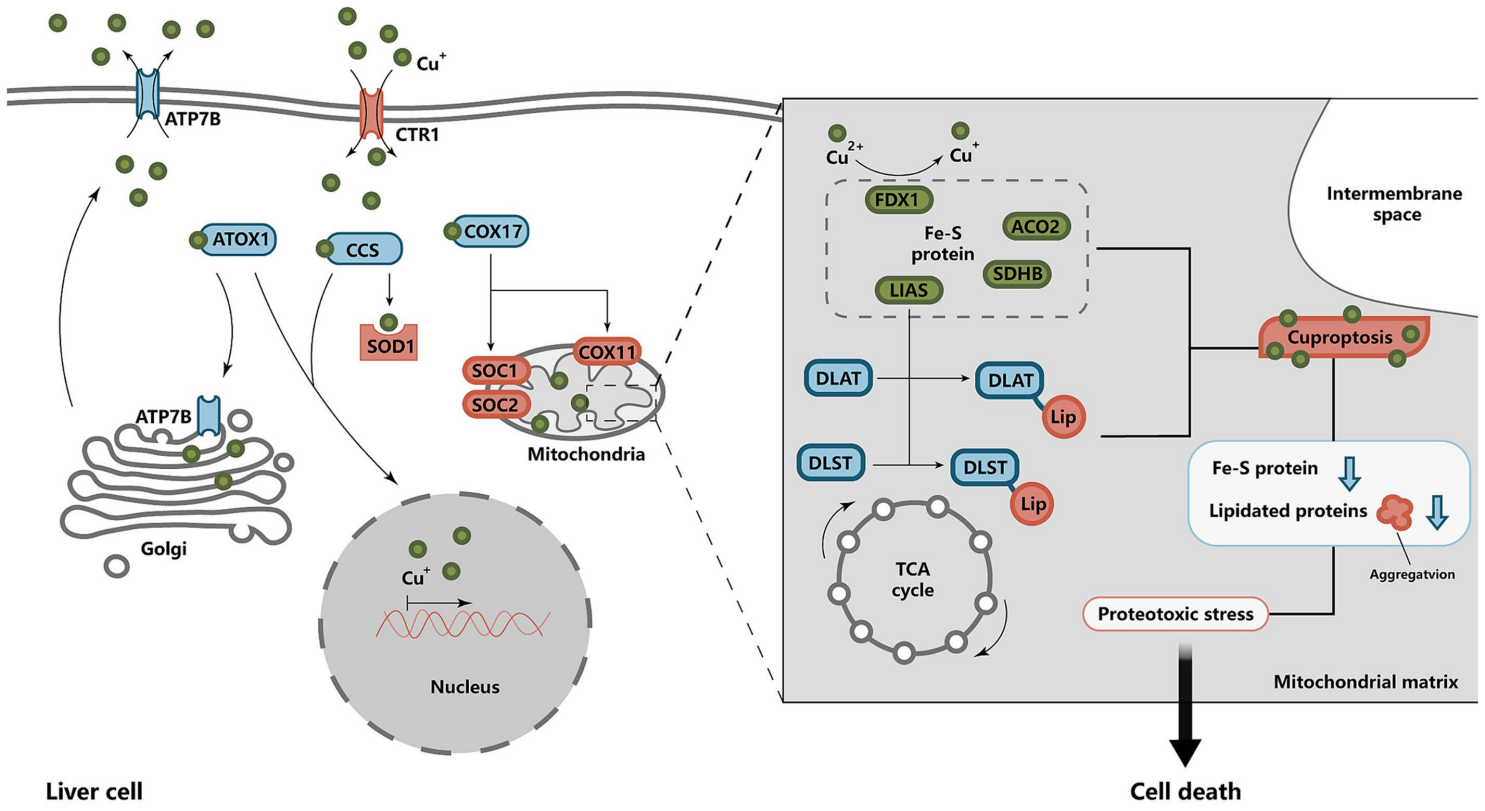
Disorder of copper metabolism in liver cells leads to cuproptosis. Hepatocytes maintain intracellular copper homeostasis through CTR1-mediated copper uptake and ATP7B-dependent copper efflux. Copper in cells is transferred to specific proteins through copper chaperones (ATOX1, CCS and COX17) to participate in biochemical reactions. Disruption of this regulatory network induces pathological copper overload in hepatocytes. Excess copper ions preferentially chelate lipoylated proteins participating in the TCA cycle, causing their quantitative depletion and pathological aggregation. This copper-mediated proteostatic disruption subsequently diminishes Fe-S cluster biogenesis, thereby eliciting proteotoxic stress that culminates in cuproptosis.

Hepatic IRI is a complex pathological process involving synergistic interactions among multiple mechanisms such as oxidative stress, calcium overload, and inflammatory cascades (26). This study is the first to reveal the critical role of cuproptosis, a newly identified copper-dependent cell death modality, in hepatic IRI. By establishing a rat hepatic IRI model, we demonstrated that IRI induces disordered copper ion metabolism in hepatocytes, leading to intracellular copper accumulation. Excessive copper ions trigger abnormal aggregation of lipoylated proteins in mitochondria and reduce Fe-S cluster proteins, ultimately resulting in cuproptosis (Figure 8). Our previous studies had confirmed that exosomes derived from ADSC-Exos can effectively mitigate mitochondrial dysfunction induced by hepatic IRI (16) and stabilize hepatocyte energy metabolism (18, 27). In this study, we are pleased to report that ADSC-Exos can effectively correct disordered copper metabolism in hepatocytes and significantly suppress IRI-induced cuproptosis, providing novel theoretical evidence for the interventional effects of exosomes in hepatic IRI.

Copper, an essential micronutrient, serves as a critical catalytic cofactor in various biological processes, including mitochondrial respiration, antioxidant responses, and biosynthesis (28). However, intracellular copper levels must be maintained within a specific range. Insufficient copper disrupts redox homeostasis and impairs normal biochemical reactions such as lipid metabolism (29), while excessive copper induces cytotoxicity and triggers cuproptosis (30). Thus, copper uptake, distribution, and excretion are tightly regulated. Hepatocytes primarily absorb copper ions transported to the liver via CTR1. These ions are either chelated with metallothionein for storage or transferred by copper chaperones to specific proteins for biochemical reactions (31). The copper chaperone ATOX1 delivers copper to ATP7B on the trans Golgi network, which subsequently exports copper out of cells via ATP7B or redistributes it to specific organelles (11). Excess systemic copper is secreted by hepatocytes into the bile and excreted through the bile ducts (32). In addition to ATOX1, CCS and COX17 are vital intracellular copper chaperones. CCS transfers copper ions to specific proteins such as SOD1 to support antioxidant responses (33). Both CCS and ATOX1 can also deliver copper to the nucleus to activate transcription factors (34). COX17 primarily transports copper to mitochondrial membrane proteins SCO1, SCO2, and COX11, enabling their roles in mitochondrial respiratory chain assembly and ensuring normal mitochondrial function (35).

In this study, we observed that hepatic IRI disrupts the copper transport system on hepatocyte membranes. The expression of CTR1 and ATP7B, both localized on the cell membrane surface, was significantly suppressed. Notably, ATP7B, as the primary protein responsible for exporting excess copper ions from hepatocytes, plays a critical role in maintaining intracellular copper homeostasis (36). Downregulation and abnormal distribution of ATP7B result in elevated intracellular copper levels in hepatocytes (37). However, it is important to note that copper does not solely act detrimentally in IRI. As a cofactor for SOD, a pivotal antioxidant metalloenzyme, maintaining appropriate copper levels in hepatocytes is essential to counteract redox imbalance triggered by IRI (38). We further examined the expression of cytoplasmic copper chaperones in hepatocytes and found that IRI markedly suppressed ATOX1, CCS, and COX17 expression. This suppression likely prevents intracellular copper ions from being properly delivered to required subcellular compartments, leading to localized copper accumulation and subsequent cytotoxicity (39). These findings collectively demonstrate that IRI simultaneously impairs copper uptake, distribution, and excretion in hepatocytes, ultimately causing intracellular copper metabolic dysregulation. Our previous studies confirmed that ADSC-Exos can alleviate hepatic IRI by modulating hepatocyte energy metabolism and improving redox imbalance (18, 25). In this study, we discovered that ADSC-Exos effectively corrects copper metabolic disorders in hepatocytes, reduces abnormal copper accumulation, and restores the copper transport system to normal levels within 3 days post-IRI. This regulatory effect may be attributed to several reasons. First, exosomes are rich in bioactive factors derived from parental cells, which enhance hepatocyte survival and accelerate metabolic system recovery after IRI (40). Moreover, studies have indicated that ADSC-Exos can effectively regulate IRI-induced endoplasmic reticulum stress (41, 42), and the endoplasmic reticulum plays an important role in intracellular copper ion metabolism via ATOX1 and ATP7B (32), which also provides support for its promotion of copper metabolism recovery in liver tissue.

As a recently identified form of cell death, cuproptosis currently lacks definitive and reliable biomarkers. Its most critical features include reduced levels of Fe-S cluster proteins and the depletion and aggregation of acylated proteins (10). We first detected the expression levels of multiple Fe-S cluster proteins and observed significant reductions in all tested proteins following IRI. Among these, FDX1 is recognized as an upstream regulator of cuproptosis. It reduces Cu^2+^ to the more cytotoxic Cu^+^, releases it into the mitochondrial matrix, and regulates protein acylation (43). Studies indicate that silencing this gene effectively suppresses protein acylation in cells and enhances resistance to cuproptosis (44). LIAS, another key enzyme regulating cuproptosis, is essential for synthesizing lipoic acid and mediates the lipoylation of TCA cycle-associated proteins (7). In contrast to these two enzymes, ACO2 primarily maintains intracellular iron pool homeostasis and mitochondrial DNA stability (45), while SDHB is a core component of mitochondrial complex II (46), with no direct involvement in cuproptosis. Another hallmark of cuproptosis involves excessive copper ions binding to lipoylated proteins in mitochondria, causing both a reduction in lipoylated protein levels and their abnormal aggregation (47). DLAT and DLST, as essential components of the mitochondrial pyruvate dehydrogenase complex and *α*-ketoglutarate dehydrogenase complex respectively, participate in aerobic respiration through their lipoylated forms (48). During IRI, hepatocytes exhibited markedly decreased total DLAT/DLST levels and reduced lipoylated DLAT levels. Immunofluorescence further revealed DLAT aggregation, collectively demonstrating that hepatic IRI induces cuproptosis in hepatocytes.

The above findings demonstrate that cuproptosis is closely linked to mitochondrial status in cells. Studies also suggest that cells with robust mitochondrial function exhibit heightened sensitivity to cuproptosis (49). As a vital detoxification and metabolic organ, the liver relies heavily on the functional integrity of abundant mitochondria within hepatocytes to sustain its physiological activities (50). However, our prior research revealed that hepatic IRI excessively activates mitophagy while suppressing mitochondrial biogenesis, ultimately leading to reduced mitochondrial content in hepatocytes (16). Notably, this absolute decrease in mitochondrial quantity caused by mitochondrial damage partially explains the observed declines in Fe-S cluster proteins and acylated proteins localized to mitochondria (51). Crucially, the abnormal aggregation of DLAT resolves this ambiguity. Excess copper ions induced by IRI bind to lipoylated DLAT, triggering this phenomenon and confirming that hepatic IRI ultimately induces cuproptosis in hepatocytes. Further investigation into the regulatory effects of ADSC-Exos on IRI induced cuproptosis revealed that exosomes effectively suppressed cuproptosis in hepatocytes and restored normal cellular status within 3 days post-IRI. This protective mechanism likely involves multiple synergistic factors. First, exosomes significantly ameliorated IRI-induced copper metabolic dysregulation in hepatocytes, thereby reducing intracellular copper ion concentrations and mitigating cuproptosis severity caused by copper overload. Second, cuproptosis is characterized by mitochondrial protein metabolic disorders that induce acute toxic protein stress, ultimately culminating in cell death (49). Previously, we found that ADSC-Exos demonstrated remarkable efficacy in alleviating mitochondrial dysfunction and reducing mitochondrial damage caused by hepatic IRI (17). This suggests that ADSC-Exos may inhibit cuproptosis resulting from hepatic IRI through this mechanism.

However, hepatic IRI is a complex pathological process involving multiple forms of cell death and modes of injury, which also interact with one another (2). For instance, during hepatic IRI, pyroptosis plays a significant role in promoting the inflammatory response (52), and it also triggers substantial ROS production, thereby affecting intracellular mitochondrial quality control and potentially influencing cuproptosis-related processes (53). Ferroptosis is considered to play a dominant role in hepatocyte injury during hepatic IRI (54), and studies have indicated that the occurrence of ferroptosis can sensitize cells to cuproptosis (22). Therefore, future research should determine the proportion of injury caused by cuproptosis in hepatic IRI and the role it plays in the crosstalk among multiple cell death modalities triggered by IRI.

In summary, we found that hepatic IRI disrupts hepatocyte copper metabolism, lead-ing to elevated intracellular copper ion levels and ultimately triggering cuproptosis in hepatocytes. ADSCs-Exo effectively ameliorate hepatic IRI-induced copper metabolic dis-orders in hepatocytes, reduce intracellular copper accumulation, and alleviate cu-proptosis. This discovery unveils a novel pathological mechanism of hepatic IRI and pro-vides fresh insights for its clinical management. Furthermore, ADSCs-Exo demonstrate the ability to regulate copper metabolic dysregulation and suppress cuproptosis caused by hepatic IRI, offering a new theoretical foundation for ADSCs-Exo-based therapeutic strate-gies against hepatic IRI. However, this study has notable limitations. Although the highly complex pathological mechanisms of hepatic IRI prompted us to investigate copper me-tabolism and cuproptosis in rat and miniature pig models, significant differences persist due to species-specific variations and discrepancies from human clinical manifestations. Due to the complex pathological processes of hepatic ischemia–reperfusion injury, this study could not fully elucidate the intricate causal relationships among hepatic IRI, copper metabolism disorder, and cuproptosis. At the same time, it must be pointed out that this study did not directly determine the ex-tent to which cuproptosis contributes to the exacerbation of hepatic IRI. Future studies employing copper chelator intervention or knockout of key proteins are warranted to es-tablish its role. Additionally, due to the complexity of ADSC-Exo cargo, we did not fully elucidate the specific molecular mechanisms by which ADSC-Exos improve copper metabolic disorders and inhibit cuproptosis in hepatic ischemia–reperfusion injury in this study. Future studies will focus on elucidating the role of cuproptosis in he-patic IRI and ad-vancing the theoretical research and clinical application of ADSCs-Exo in liver injury management.

## 5 Conclusion

This study is the first to elucidate a novel mechanism by which hepatic IRI triggers cuproptosis through disrupting copper metabolism in hepatocytes. IRI not only disrupts the expression of membrane surface proteins such as CTR1 and ATP7B, impairing copper ion uptake and efflux, but also suppresses copper chaperone expression, thereby compromising intracellular copper transport and ultimately inducing a cuproptosis cascade. Notably, ADSCs-Exo exhibits unique regulatory effects, effectively correcting copper metabolic dysregulation in hepatocytes, reversing copper overload, and halting cuproptosis. These findings not only provide a groundbreaking perspective for deciphering the pathological progression of IRI but also reveal that ADSCs-Exo exerts hepatoprotective effects by modulating copper homeostasis to inhibit cuproptosis, offering innovative therapeutic strategies for clinical prevention of liver transplantation-associated injuries.

## Data Availability

The original contributions presented in the study are included in the article/Supplementary material, further inquiries can be directed to the corresponding author.
