# Supplementary material for: Adipose-derived mesenchymal stem cell exosomes ameliorate copper metabolism dysregulation and reduce cuproptosis caused by liver IRI
Source: Front Vet Sci. 2026 Jul 15;13:1895340. doi: 10.3389/fvets.2026.1895340 (PMC13414201; doi:10.3389/fvets.2026.1895340)

Original image of CTR1 expression in rat tissues detected by Western blot

<table>
<tr><td colspan="3">CTR1-rat</td></tr>
<tr><td>Repeat 1</td><td>Repeat 2</td><td>Repeat 3</td></tr>
<tr><td>Sham IRI</td><td>Sham IRI</td><td>Sham IRI</td></tr>
<tr><td>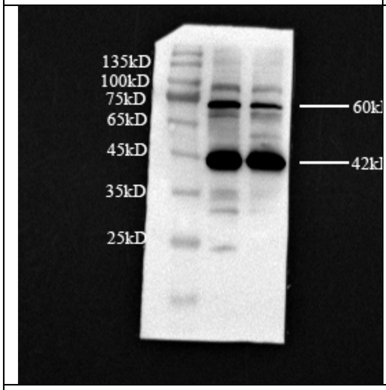
</td><td>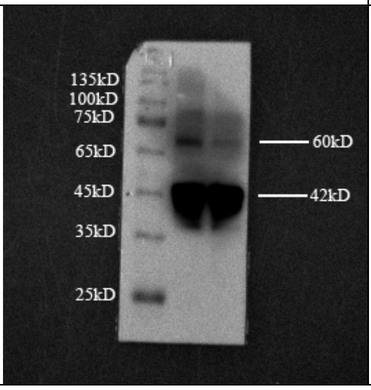
</td><td>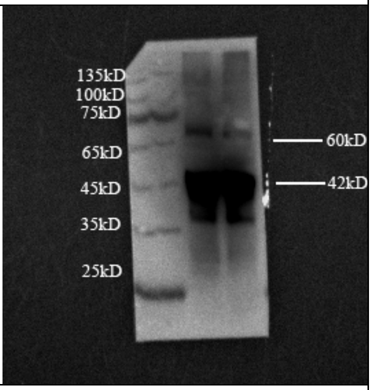
</td></tr>
<tr><td colspan="3">actin-rat</td></tr>
<tr><td>Repeat 1</td><td>Repeat 2</td><td>Repeat 3</td></tr>
<tr><td>Sham IRI</td><td>Sham IRI</td><td>Sham IRI</td></tr>
<tr><td>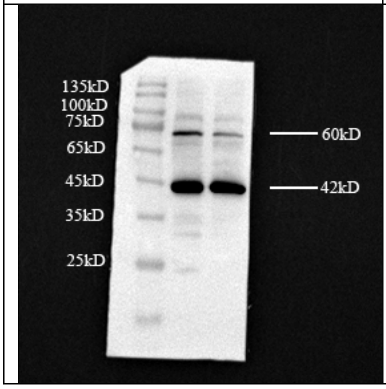
</td><td>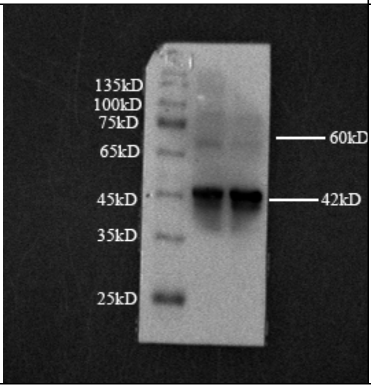
</td><td>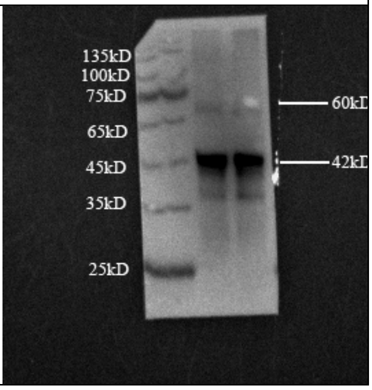
</td></tr>
</table>

Original image of ATP7B expression in rat tissues detected by Western blot

<table>
<tr><td colspan="3">ATP7B-rat</td></tr>
<tr><td>Repeat 1</td><td>Repeat 2</td><td>Repeat 3</td></tr>
<tr><td>Sham IRI</td><td>Sham IRI</td><td>Sham IRI</td></tr>
<tr><td>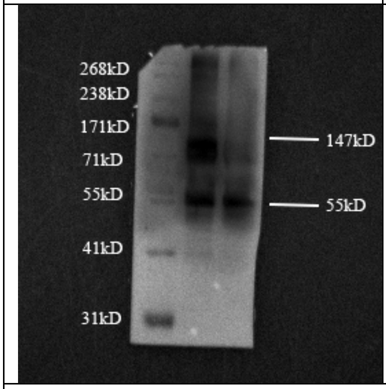
</td><td>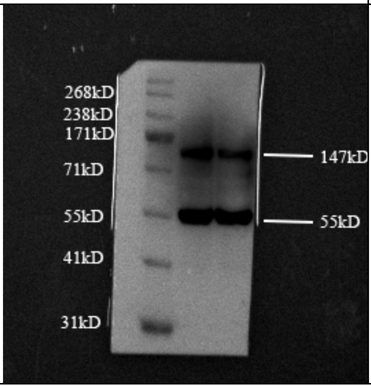
</td><td>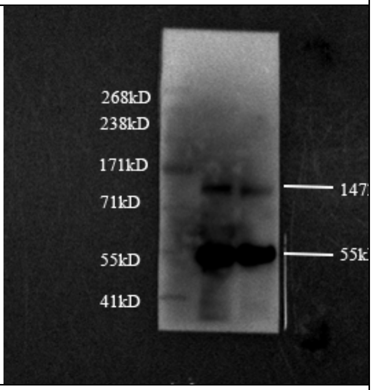
</td></tr>
<tr><td colspan="3">tubulin-rat</td></tr>
<tr><td>Repeat 1</td><td>Repeat 2</td><td>Repeat 3</td></tr>
<tr><td>Sham IRI</td><td>Sham IRI</td><td>Sham IRI</td></tr>
<tr><td>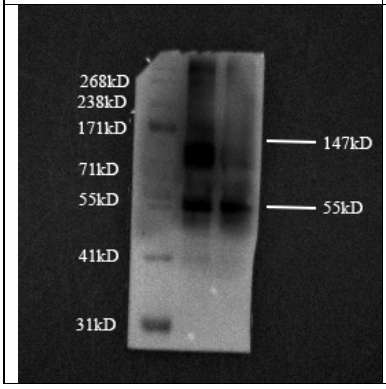
</td><td>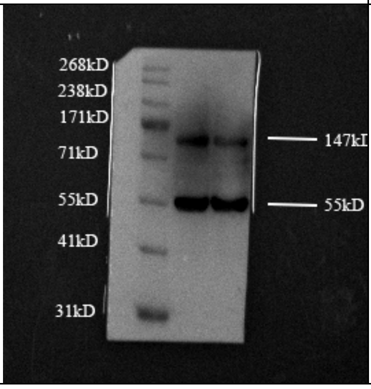
</td><td>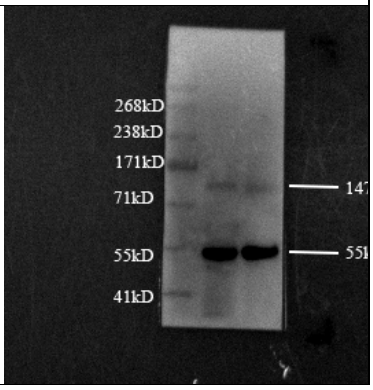
</td></tr>
</table>

Original image of FDX1 expression in rat tissues detected by Western blot

<table>
<tr><td colspan="3">FDX1-rat</td></tr>
<tr><td>Repeat 1</td><td>Repeat 2</td><td>Repeat 3</td></tr>
<tr><td>Sham IRI</td><td>Sham IRI</td><td>Sham IRI</td></tr>
<tr><td>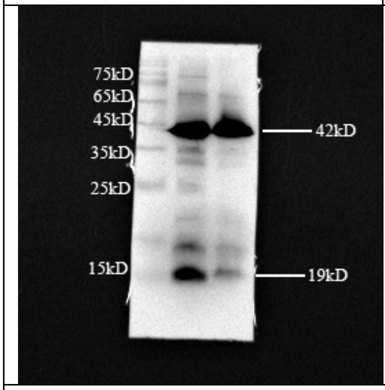</td><td>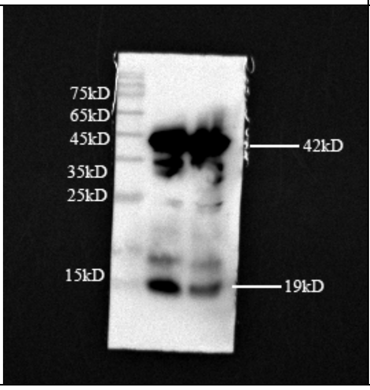</td><td>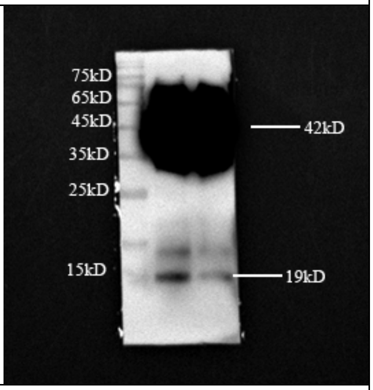</td></tr>
<tr><td colspan="3">actin-rat</td></tr>
<tr><td>Repeat 1</td><td>Repeat 2</td><td>Repeat 3</td></tr>
<tr><td>Sham IRI</td><td>Sham IRI</td><td>Sham IRI</td></tr>
<tr><td>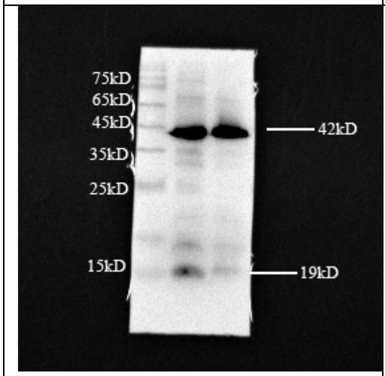</td><td>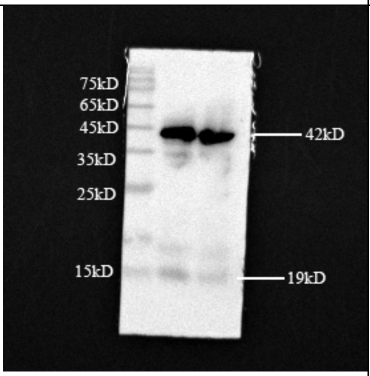</td><td>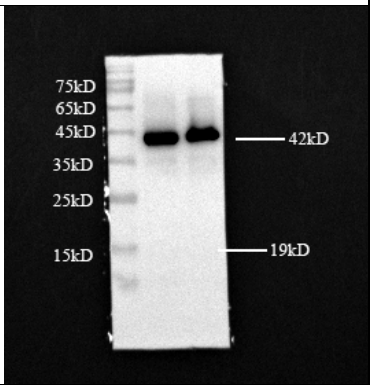</td></tr>
</table>

Original image of LIAS expression in rat tissues detected by Western blot

<table>
<tr><td colspan="3">LIAS-rat</td></tr>
<tr><td>Repeat 1</td><td>Repeat 2</td><td>Repeat 3</td></tr>
<tr><td>Sham IRI</td><td>Sham IRI</td><td>Sham IRI</td></tr>
<tr><td>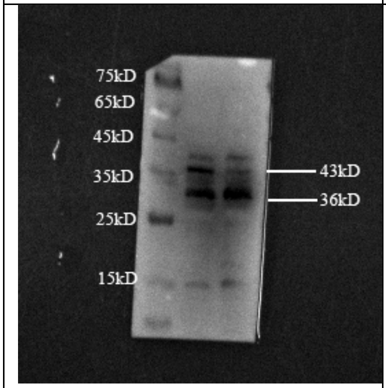</td><td>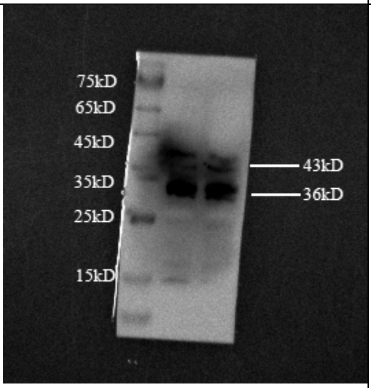</td><td>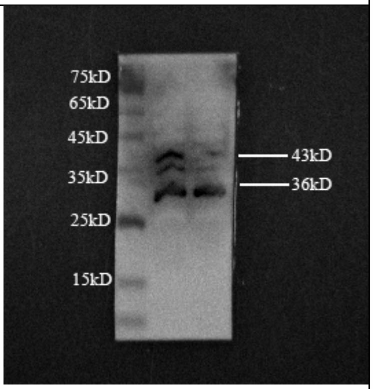</td></tr>
<tr><td colspan="3">GAPDH-rat</td></tr>
<tr><td>Repeat 1</td><td>Repeat 2</td><td>Repeat 3</td></tr>
<tr><td>Sham IRI</td><td>Sham IRI</td><td>Sham IRI</td></tr>
<tr><td>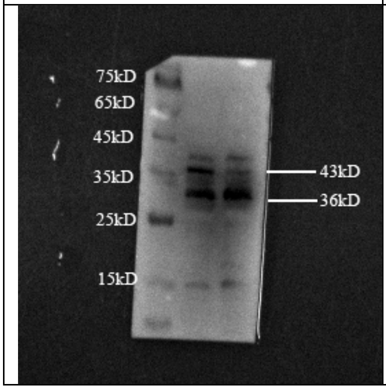</td><td>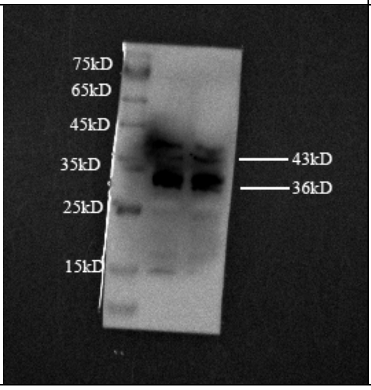</td><td>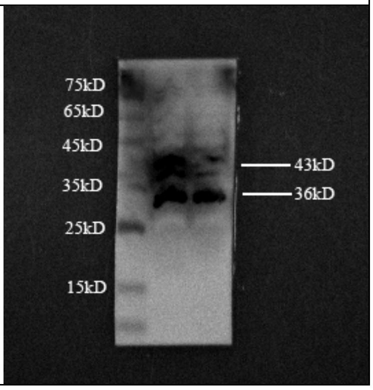</td></tr>
</table>

Original image of ACO2 expression in rat tissues detected by Western blot

<table>
<tr><td colspan="3">ACO2-rat</td></tr>
<tr><td>Repeat 1</td><td>Repeat 2</td><td>Repeat 3</td></tr>
<tr><td>Sham IRI</td><td>Sham IRI</td><td>Sham IRI</td></tr>
<tr>
<td>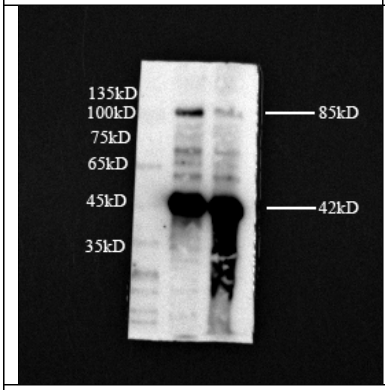
</td>
<td>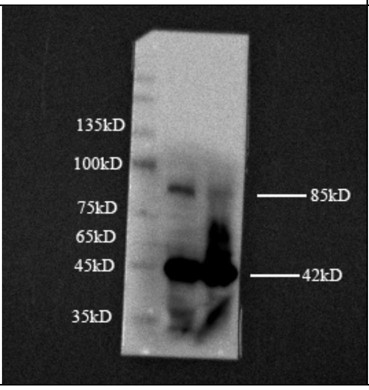
</td>
<td>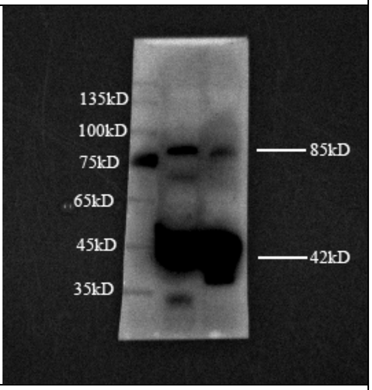
</td>
</tr>
<tr><td colspan="3">actin-rat</td></tr>
<tr><td>Repeat 1</td><td>Repeat 2</td><td>Repeat 3</td></tr>
<tr><td>Sham IRI</td><td>Sham IRI</td><td>Sham IRI</td></tr>
<tr>
<td>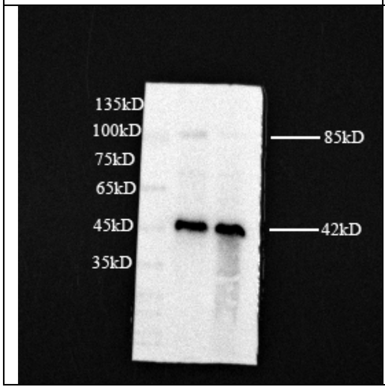
</td>
<td>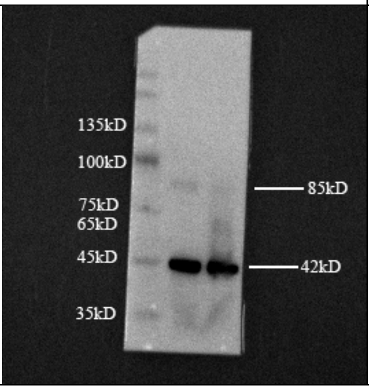
</td>
<td>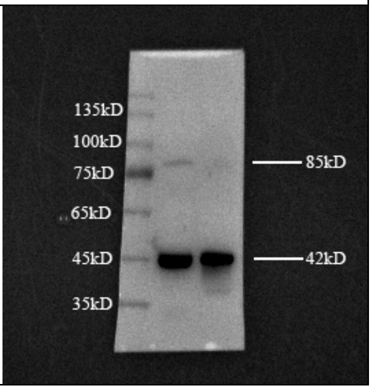
</td>
</tr>
</table>

Original image of SDHB expression in rat tissues detected by Western blot

SDHB-rat		
Repeat 1	Repeat 2	Repeat 3
Sham IRI	Sham IRI	Sham IRI
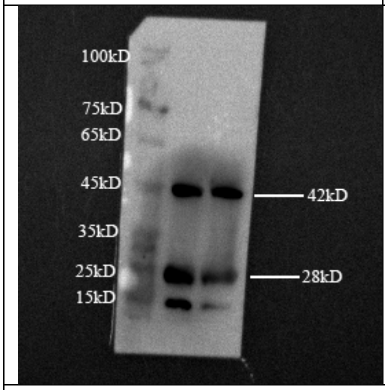	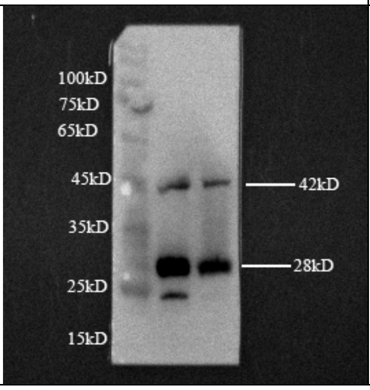	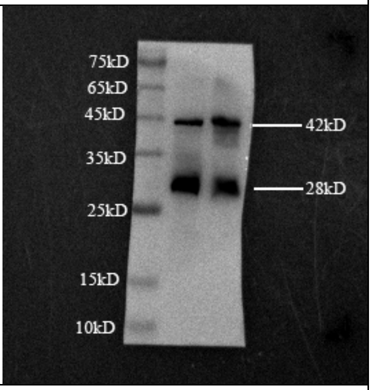
actin-rat		
Repeat 1	Repeat 2	Repeat 3
Sham IRI	Sham IRI	Sham IRI
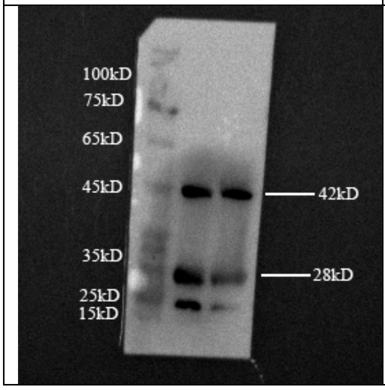	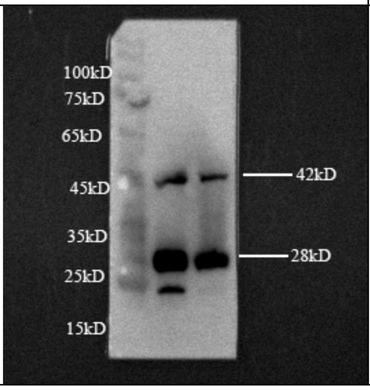	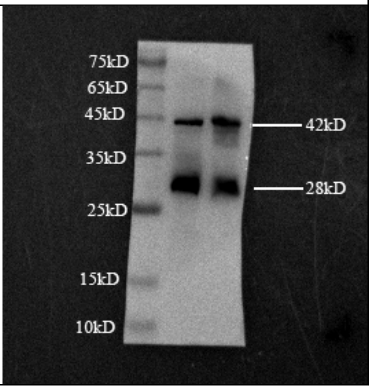

Original image of HSP70 expression in rat tissues detected by Western blot

HSP70-rat		
Repeat 1	Repeat 2	Repeat 3
Sham IRI	Sham IRI	Sham IRI
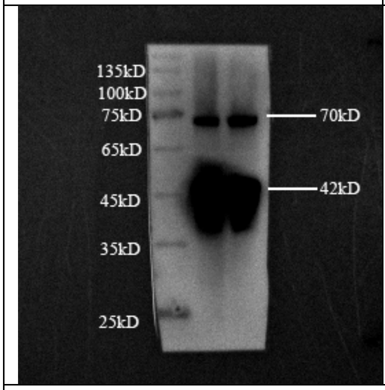	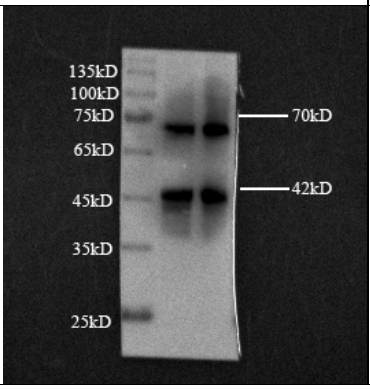	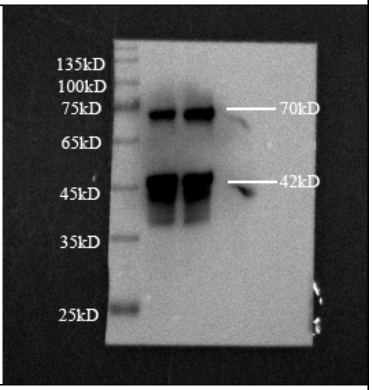
actin-rat		
Repeat 1	Repeat 2	Repeat 3
Sham IRI	Sham IRI	Sham IRI
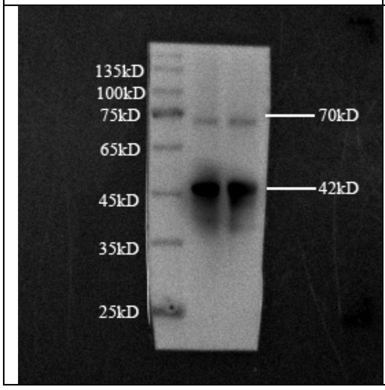	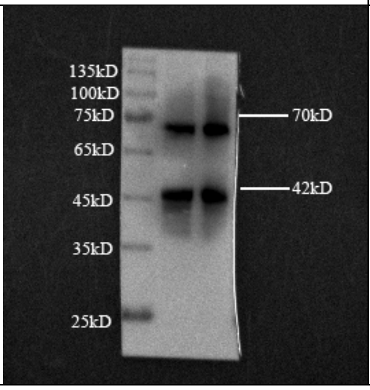	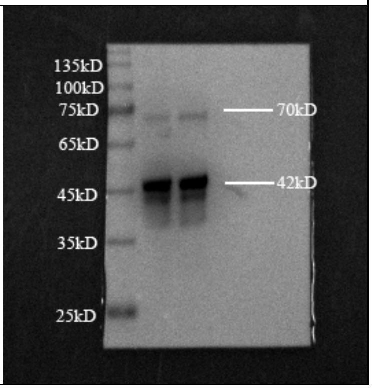

Original image of Lip-DLAT expression in rat tissues detected by Western blot

<table>
<tr><td colspan="3">Lip-DLAT-rat</td></tr>
<tr><td>Repeat 1</td><td>Repeat 2</td><td>Repeat 3</td></tr>
<tr><td>Sham IRI</td><td>Sham IRI</td><td>Sham IRI</td></tr>
<tr><td>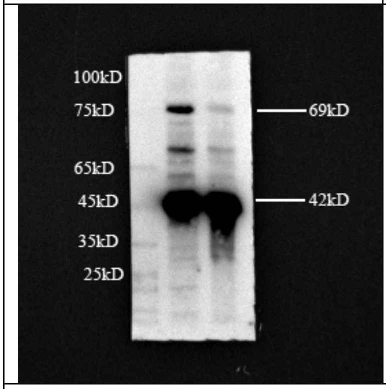
</td><td>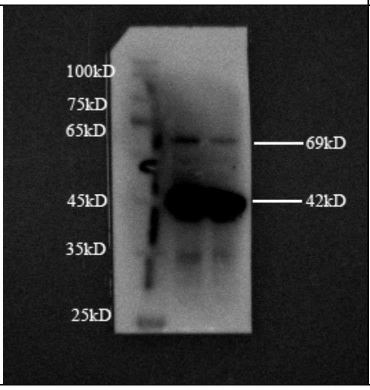
</td><td>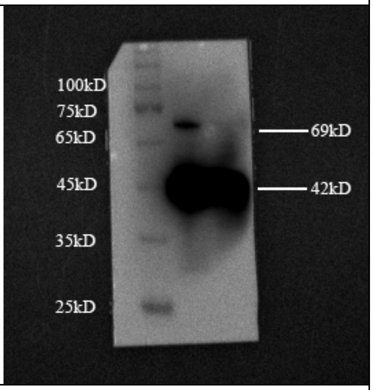
</td></tr>
<tr><td colspan="3">actin-rat</td></tr>
<tr><td>Repeat 1</td><td>Repeat 2</td><td>Repeat 3</td></tr>
<tr><td>Sham IRI</td><td>Sham IRI</td><td>Sham IRI</td></tr>
<tr><td>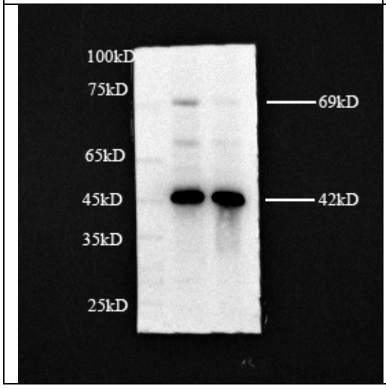
</td><td>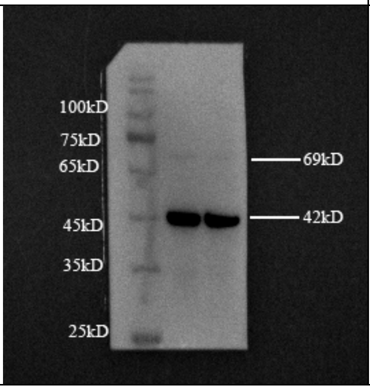
</td><td>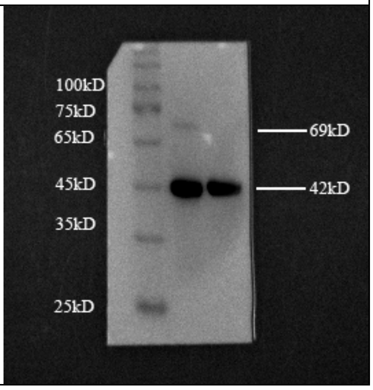
</td></tr>
</table>

Original image of DLAT expression in rat tissues detected by Western blot

DLAT-rat		
Repeat 1	Repeat 2	Repeat 3
Sham IRI	Sham IRI	Sham IRI
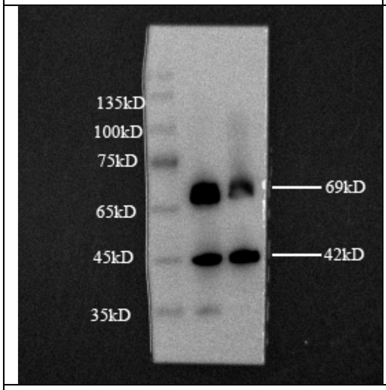	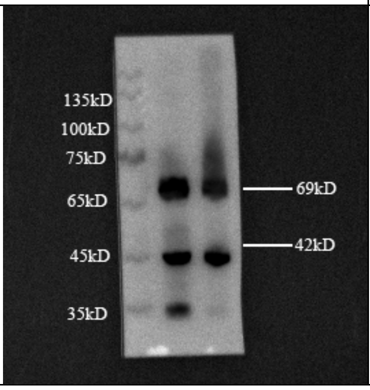	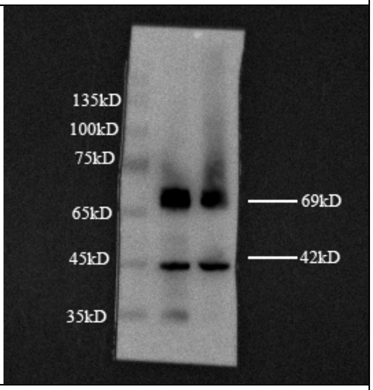
actin-rat		
Repeat 1	Repeat 2	Repeat 3
Sham IRI	Sham IRI	Sham IRI
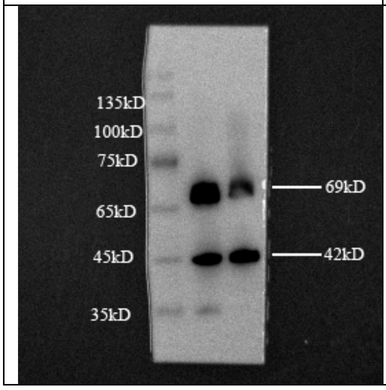	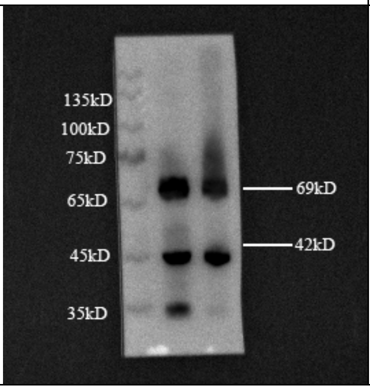	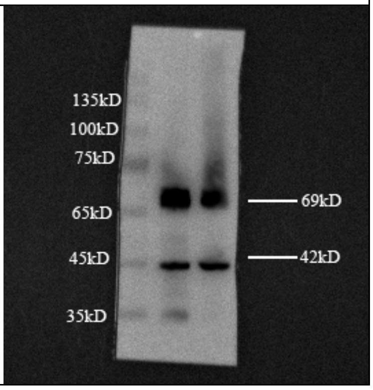

Original image of DLST expression in rat tissues detected by Western blot

<table>
<tr><td colspan="3">DLST-rat</td></tr>
<tr><td>Repeat 1</td><td>Repeat 2</td><td>Repeat 3</td></tr>
<tr><td>Sham IRI</td><td>Sham IRI</td><td>Sham IRI</td></tr>
<tr><td>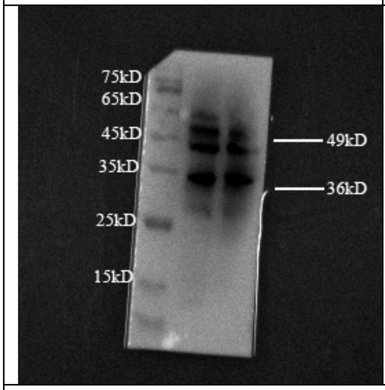
</td><td>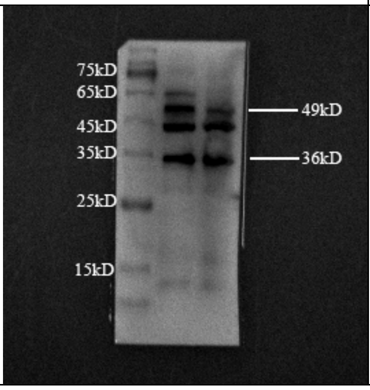
</td><td>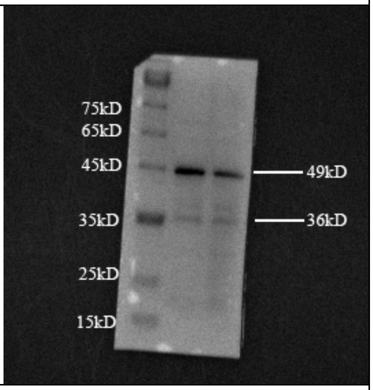
</td></tr>
<tr><td colspan="3">GAPDH-rat</td></tr>
<tr><td>Repeat 1</td><td>Repeat 2</td><td>Repeat 3</td></tr>
<tr><td>Sham IRI</td><td>Sham IRI</td><td>Sham IRI</td></tr>
<tr><td>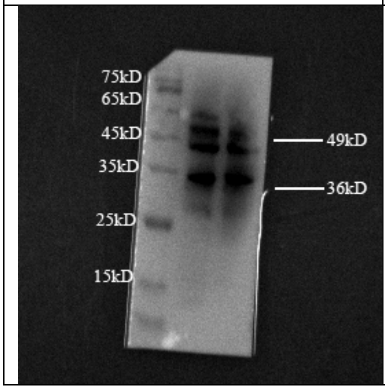
</td><td>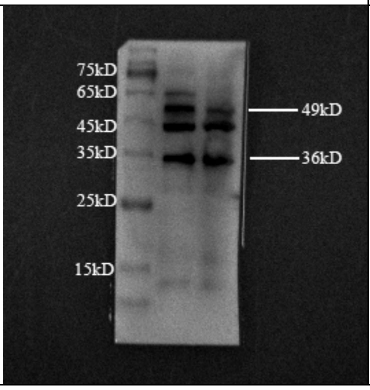
</td><td>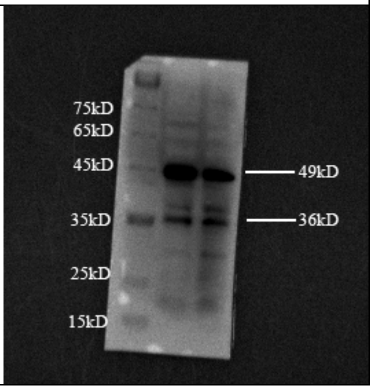
</td></tr>
</table>

Original image of CTR1 expression in pig tissues detected by Western blot

CTR1-pig-1d		
Repeat 1	Repeat 2	Repeat 3
Sham IRI Exo	Sham IRI Exo	Sham IRI Exo
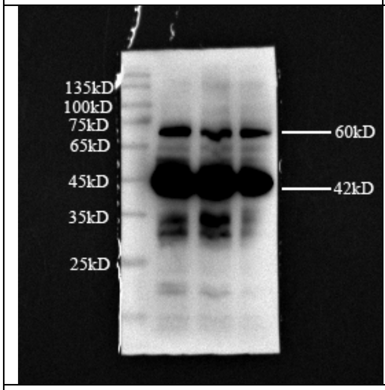	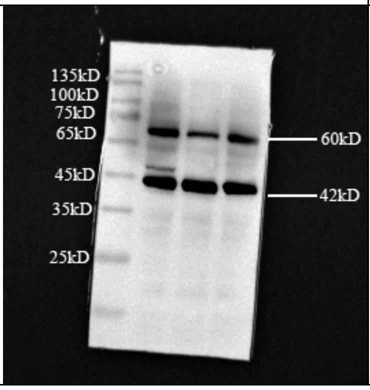	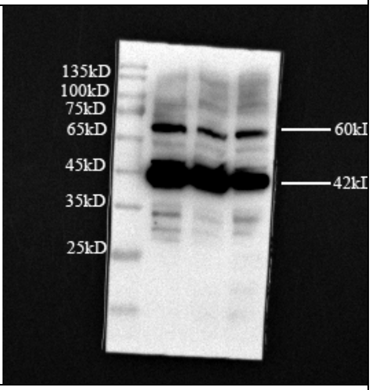

actin-pig-1d		
Repeat 1	Repeat 2	Repeat 3
Sham IRI Exo	Sham IRI Exo	Sham IRI Exo
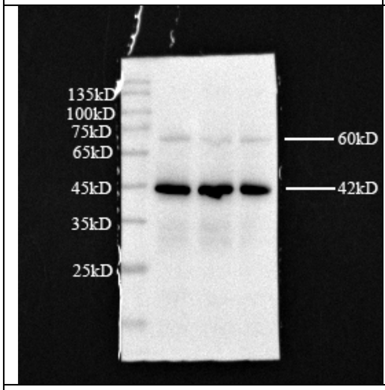	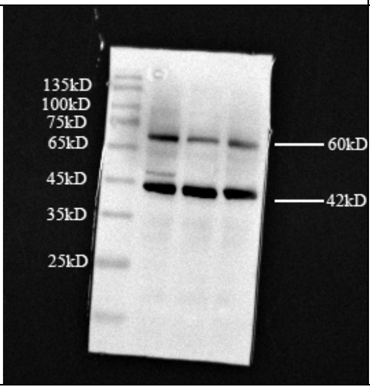	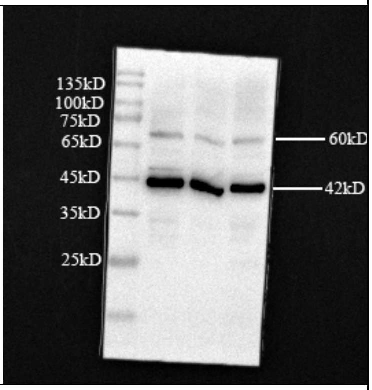

CTR1-pig-3d		
Repeat 1	Repeat 1	Repeat 1
Sham IRI Exo	Sham IRI Exo	Sham IRI Exo
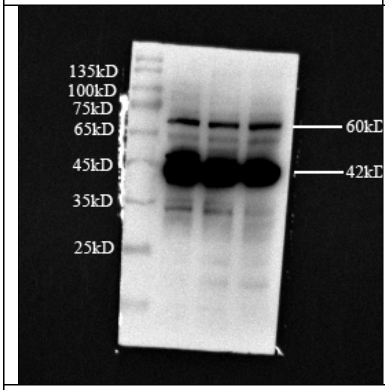	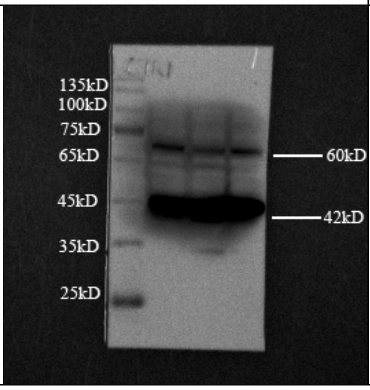	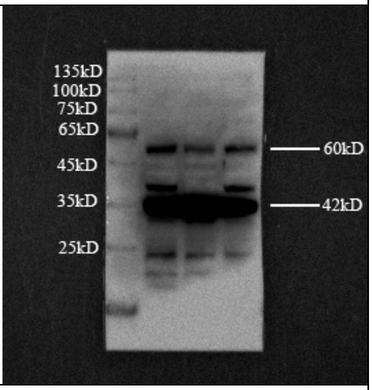

actin-pig-3d		
Repeat 1	Repeat 1	Repeat 1
Sham IRI Exo	Sham IRI Exo	Sham IRI Exo
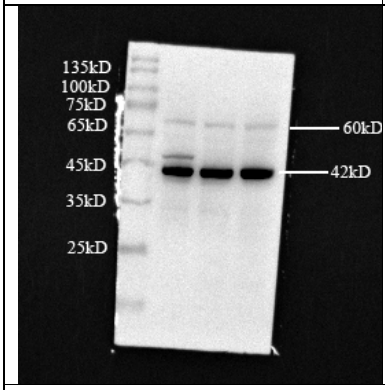	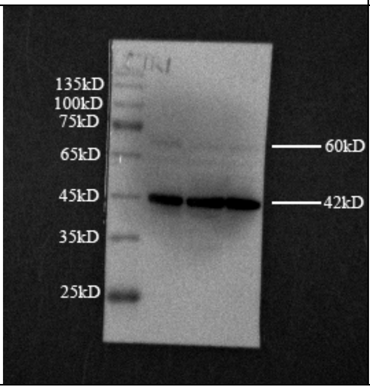	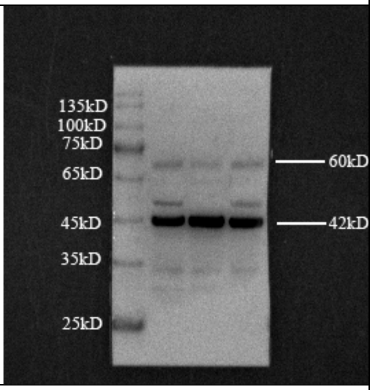
CTR1-pig-7d		
Repeat 1	Repeat 1	Repeat 1
Sham IRI Exo	Sham IRI Exo	Sham IRI Exo
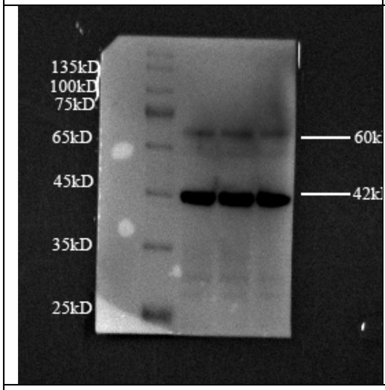	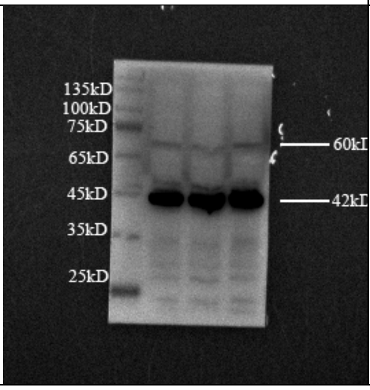	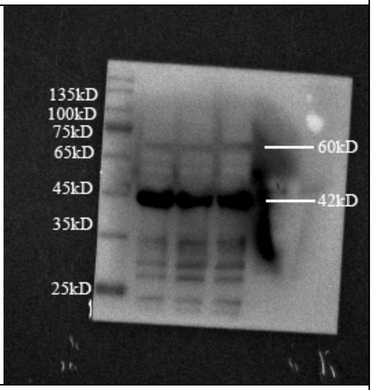
actin-pig-7d		
Repeat 1	Repeat 1	Repeat 1
Sham IRI Exo	Sham IRI Exo	Sham IRI Exo
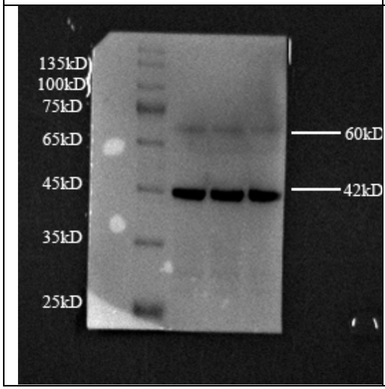	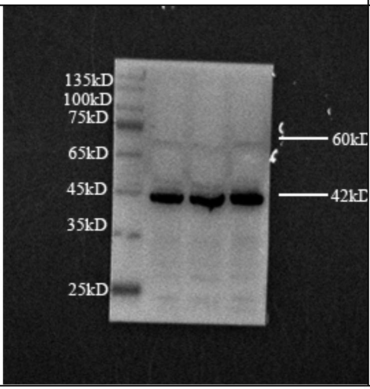	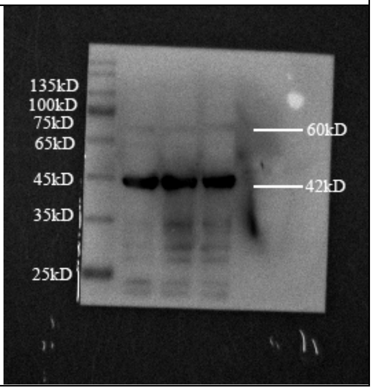

Original image of ATP7B expression in pig tissues detected by Western blot

<table>
<tr><td colspan="3">ATP7B-pig-1d</td></tr>
<tr><td>Repeat 1</td><td>Repeat 2</td><td>Repeat 3</td></tr>
<tr><td>Sham IRI Exo</td><td>Sham IRI Exo</td><td>Sham IRI Exo</td></tr>
<tr><td>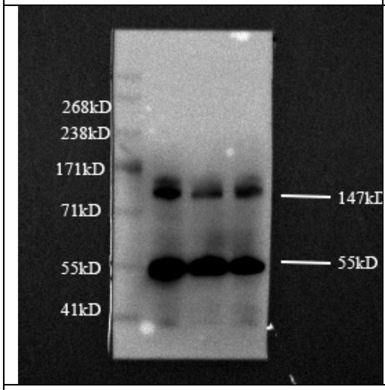
</td><td>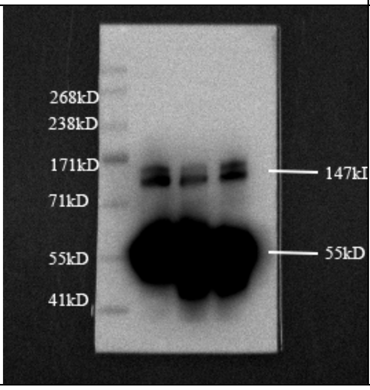
</td><td>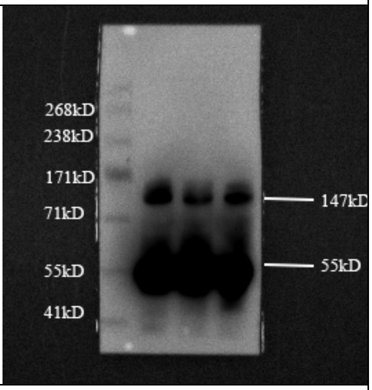
</td></tr>
<tr><td colspan="3">tubulin-pig-1d</td></tr>
<tr><td>Repeat 1</td><td>Repeat 2</td><td>Repeat 3</td></tr>
<tr><td>Sham IRI Exo</td><td>Sham IRI Exo</td><td>Sham IRI Exo</td></tr>
<tr><td>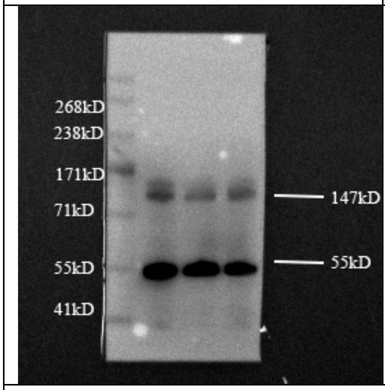
</td><td>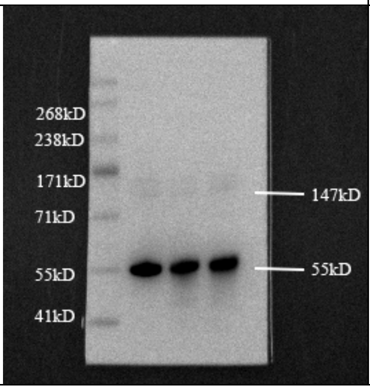
</td><td>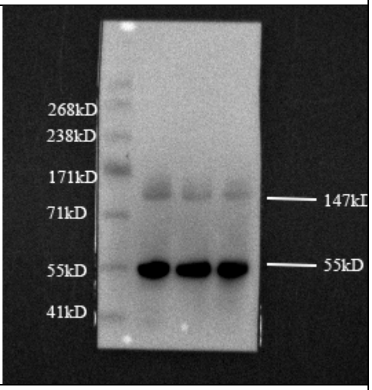
</td></tr>
<tr><td colspan="3">ATP7B-pig-3d</td></tr>
<tr><td>Repeat 1</td><td>Repeat 1</td><td>Repeat 1</td></tr>
<tr><td>Sham IRI Exo</td><td>Sham IRI Exo</td><td>Sham IRI Exo</td></tr>
<tr><td>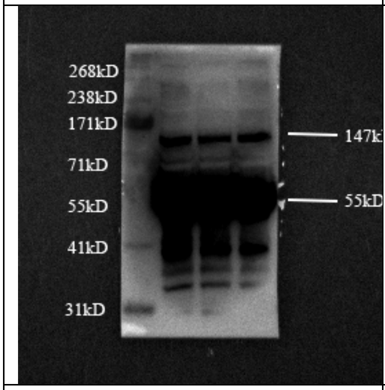
</td><td>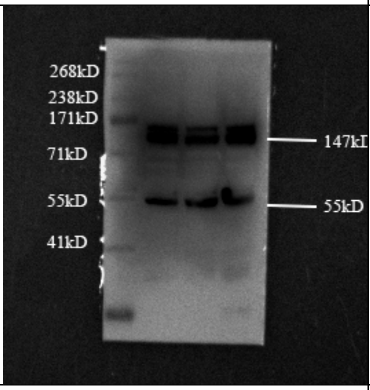
</td><td>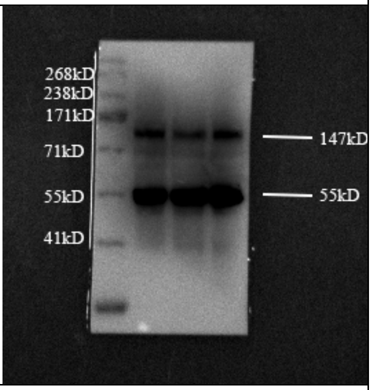
</td></tr>
<tr><td></td><td></td><td></td></tr>
</table>

tubulin-pig-3d		
Repeat 1	Repeat 1	Repeat 1
Sham IRI Exo	Sham IRI Exo	Sham IRI Exo

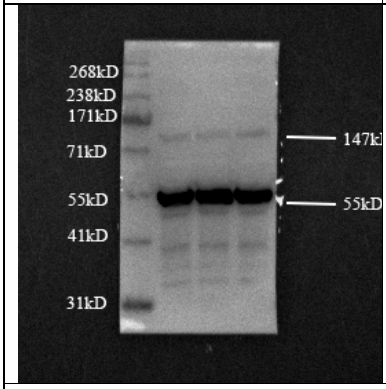

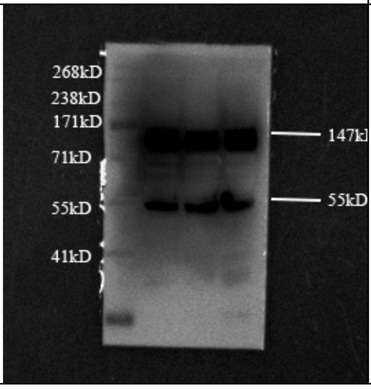

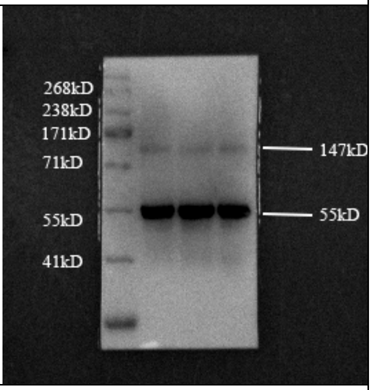

ATP7B-pig-7d		
Repeat 1	Repeat 1	Repeat 1
Sham IRI Exo	Sham IRI Exo	Sham IRI Exo

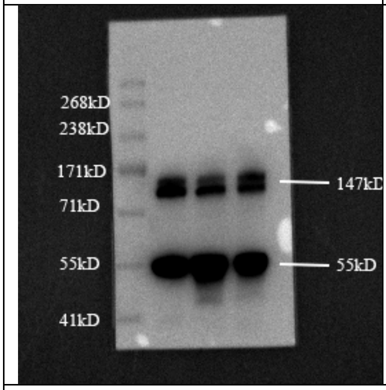

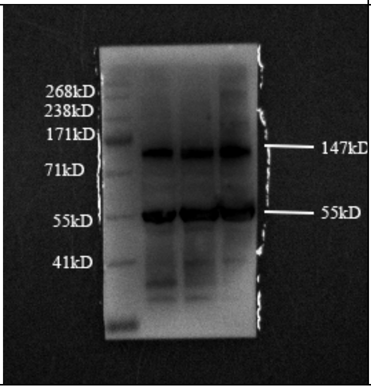

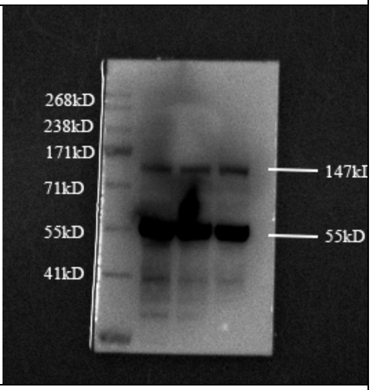

tubulin-pig-7d		
Repeat 1	Repeat 1	Repeat 1
Sham IRI Exo	Sham IRI Exo	Sham IRI Exo

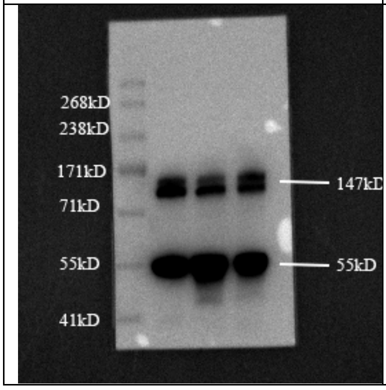

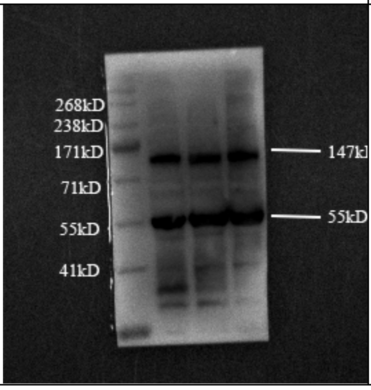

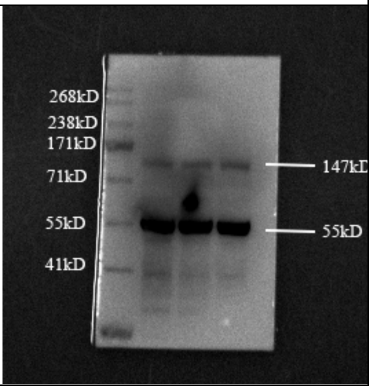

Original image of FDX1 expression in pig tissues detected by Western blot

<table>
<tr><td colspan="3">FDX1-pig-1d</td></tr>
<tr><td>Repeat 1</td><td>Repeat 2</td><td>Repeat 3</td></tr>
<tr><td>Sham IRI Exo</td><td>Sham IRI Exo</td><td>Sham IRI Exo</td></tr>
<tr><td>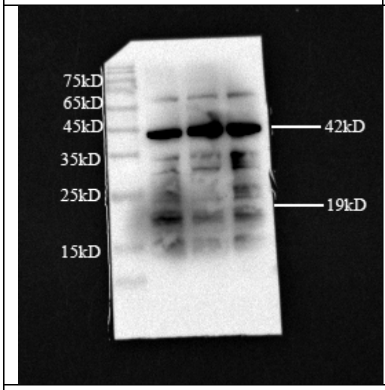</td><td>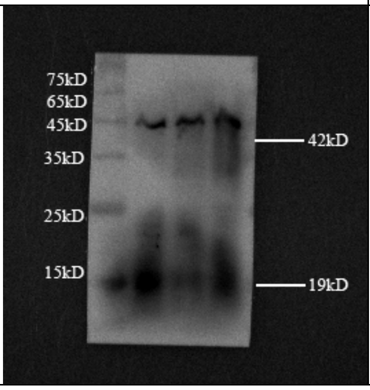</td><td>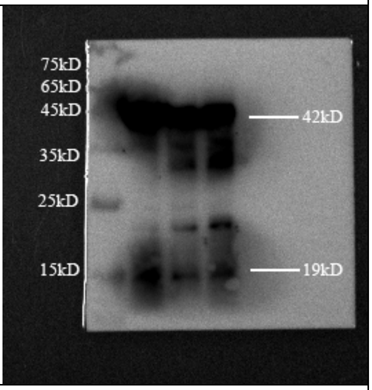</td></tr>
<tr><td colspan="3">actin-pig-1d</td></tr>
<tr><td>Repeat 1</td><td>Repeat 2</td><td>Repeat 3</td></tr>
<tr><td>Sham IRI Exo</td><td>Sham IRI Exo</td><td>Sham IRI Exo</td></tr>
<tr><td>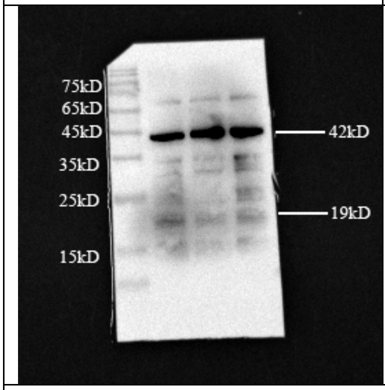</td><td>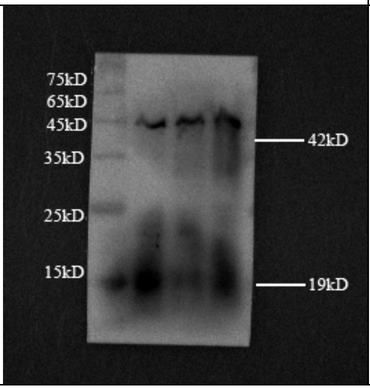</td><td>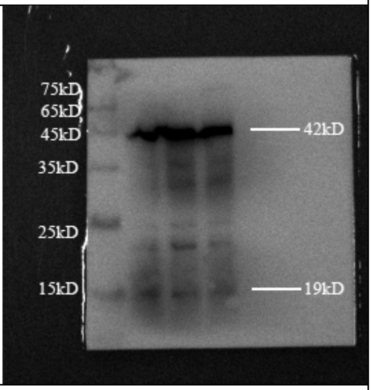</td></tr>
<tr><td colspan="3">FDX1-pig-3d</td></tr>
<tr><td>Repeat 1</td><td>Repeat 1</td><td>Repeat 1</td></tr>
<tr><td>Sham IRI Exo</td><td>Sham IRI Exo</td><td>Sham IRI Exo</td></tr>
<tr><td>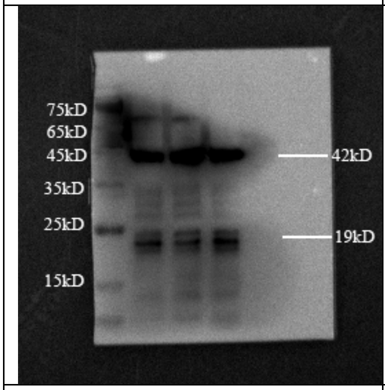</td><td>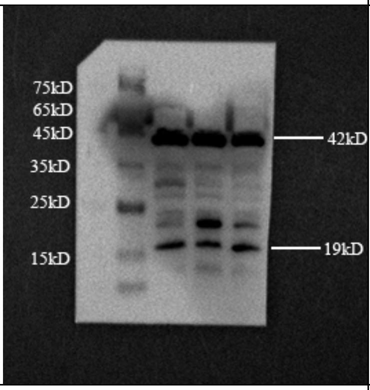</td><td>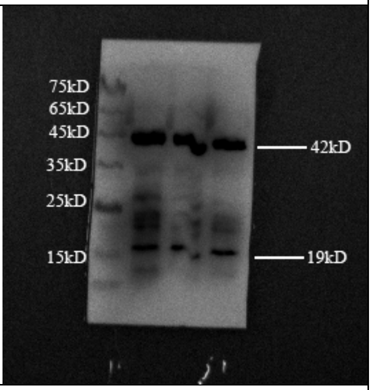</td></tr>
<tr><td></td><td></td><td></td></tr>
</table>

<table>
<tr><td colspan="3">actin-pig-3d</td></tr>
<tr><td>Repeat 1</td><td>Repeat 1</td><td>Repeat 1</td></tr>
<tr><td>Sham IRI Exo</td><td>Sham IRI Exo</td><td>Sham IRI Exo</td></tr>
<tr><td>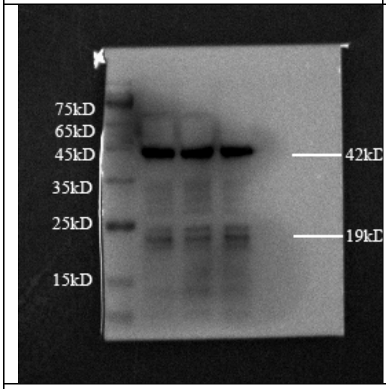</td><td>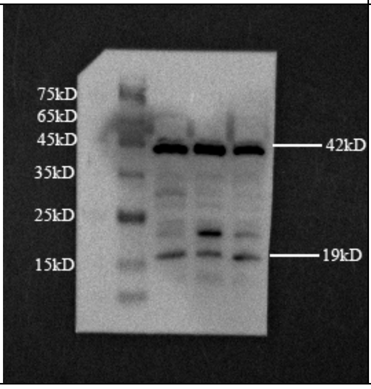</td><td>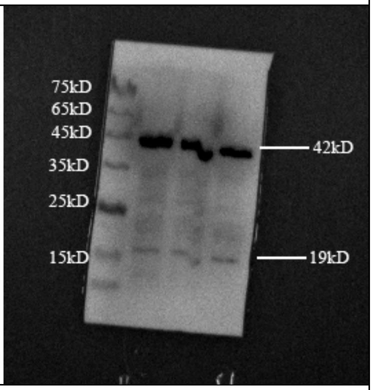</td></tr>
<tr><td colspan="3">FDX1-pig-7d</td></tr>
<tr><td>Repeat 1</td><td>Repeat 1</td><td>Repeat 1</td></tr>
<tr><td>Sham IRI Exo</td><td>Sham IRI Exo</td><td>Sham IRI Exo</td></tr>
<tr><td>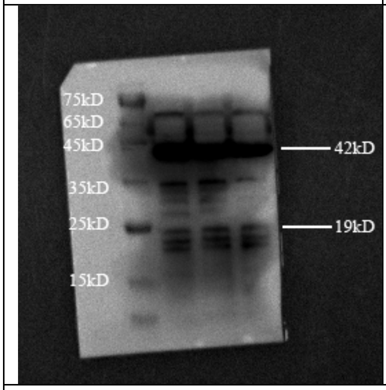</td><td>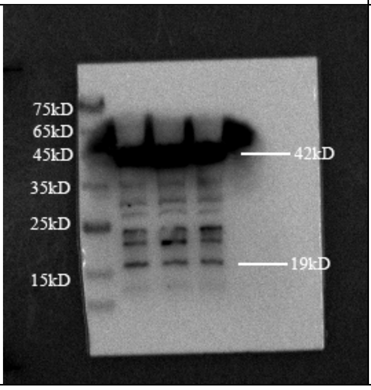</td><td>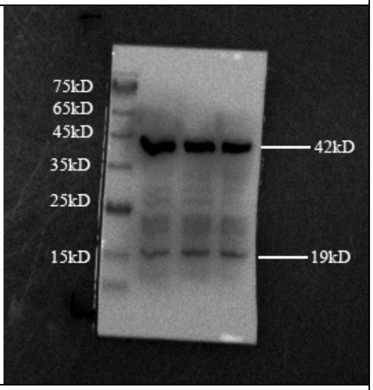</td></tr>
<tr><td colspan="3">actin-pig-7d</td></tr>
<tr><td>Repeat 1</td><td>Repeat 1</td><td>Repeat 1</td></tr>
<tr><td>Sham IRI Exo</td><td>Sham IRI Exo</td><td>Sham IRI Exo</td></tr>
<tr><td>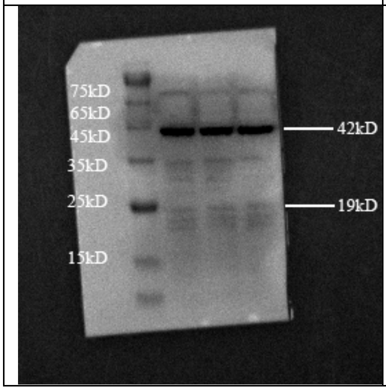</td><td>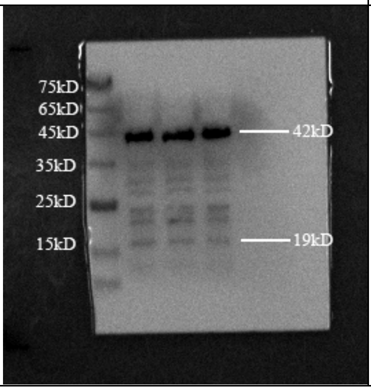</td><td>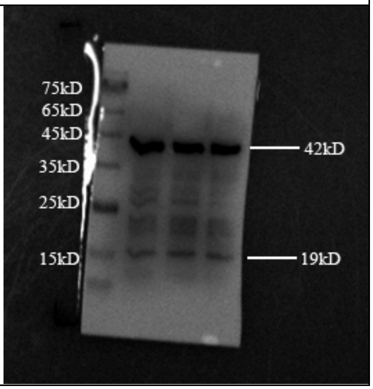</td></tr>
</table>

Original image of LIAS expression in pig tissues detected by Western blot

<table>
<tr><td colspan="3">LIAS-pig-1d</td></tr>
<tr><td>Repeat 1</td><td>Repeat 2</td><td>Repeat 3</td></tr>
<tr><td>Sham IRI Exo</td><td>Sham IRI Exo</td><td>Sham IRI Exo</td></tr>
<tr><td>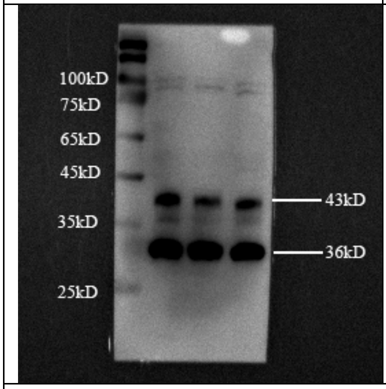
</td><td>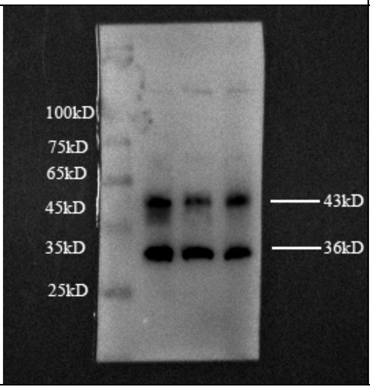
</td><td>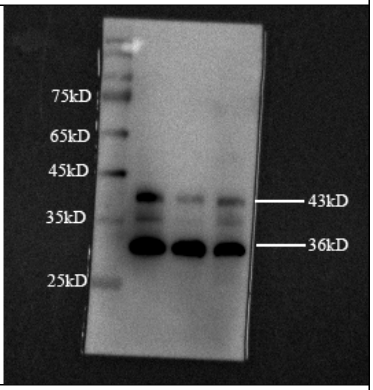
</td></tr>
<tr><td colspan="3">GAPDH-pig-1d</td></tr>
<tr><td>Repeat 1</td><td>Repeat 2</td><td>Repeat 3</td></tr>
<tr><td>Sham IRI Exo</td><td>Sham IRI Exo</td><td>Sham IRI Exo</td></tr>
<tr><td>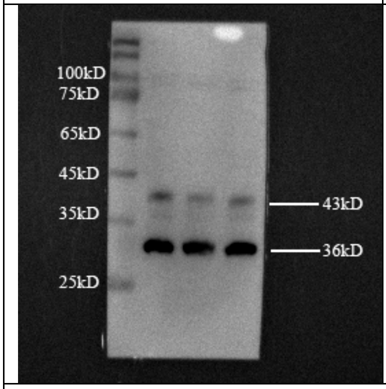
</td><td>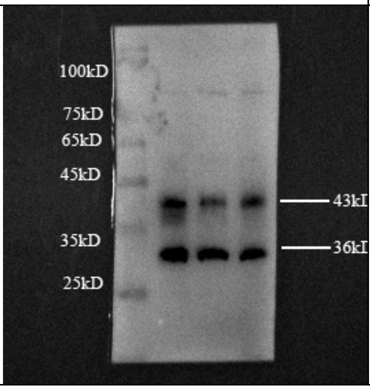
</td><td>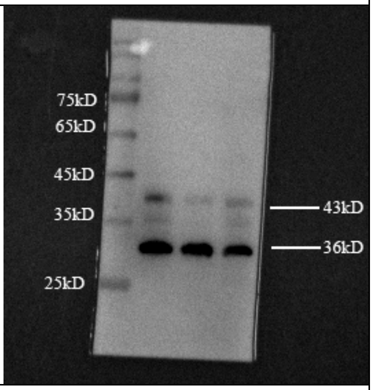
</td></tr>
<tr><td colspan="3">LIAS-pig-3d</td></tr>
<tr><td>Repeat 1</td><td>Repeat 1</td><td>Repeat 1</td></tr>
<tr><td>Sham IRI Exo</td><td>Sham IRI Exo</td><td>Sham IRI Exo</td></tr>
<tr><td>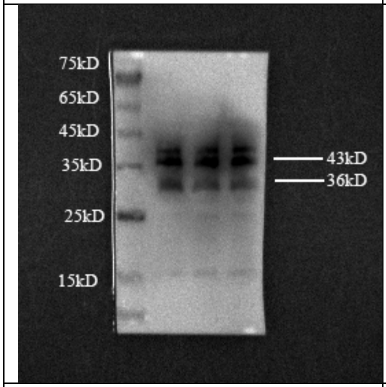
</td><td>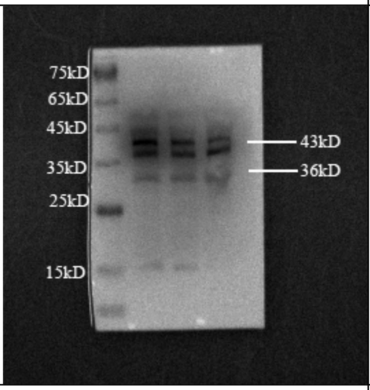
</td><td>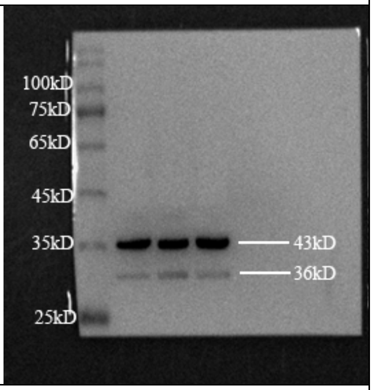
</td></tr>
<tr><td></td><td></td><td></td></tr>
</table>

GAPDH-pig-3d

Repeat 1	Repeat 1	Repeat 1
Sham IRI Exo	Sham IRI Exo	Sham IRI Exo

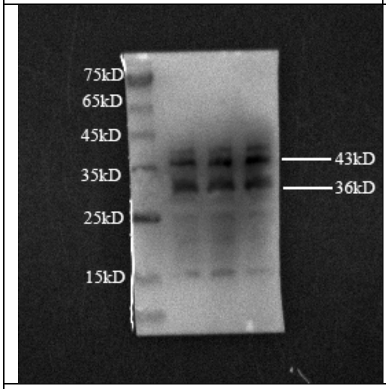

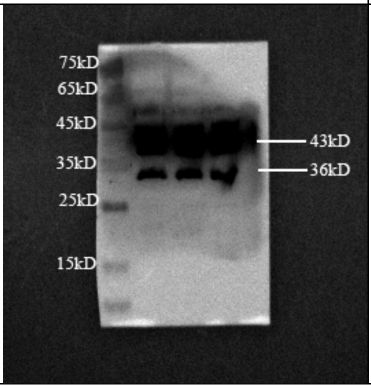

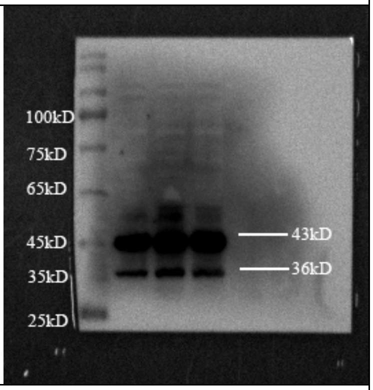

LIAS-pig-7d

Repeat 1	Repeat 1	Repeat 1
Sham IRI Exo	Sham IRI Exo	Sham IRI Exo

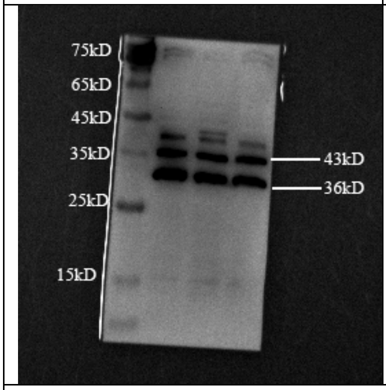

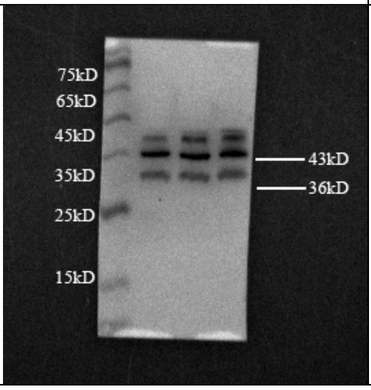

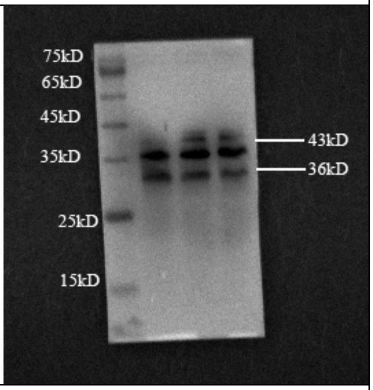

GAPDH-pig-7d

Repeat 1	Repeat 1	Repeat 1
Sham IRI Exo	Sham IRI Exo	Sham IRI Exo

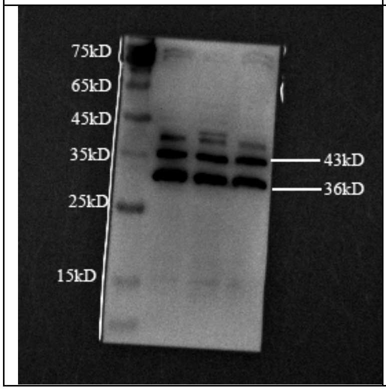

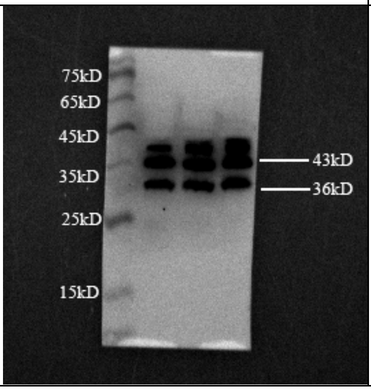

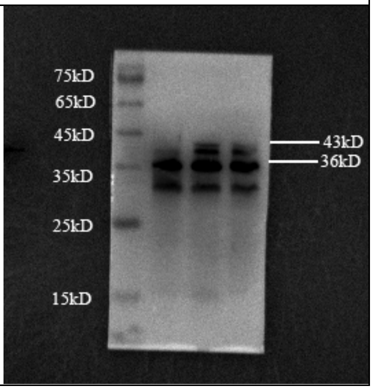

Original image of ACO2 expression in pig tissues detected by Western blot

ACO2-pig-1d		
Repeat 1	Repeat 2	Repeat 3
Sham IRI Exo	Sham IRI Exo	Sham IRI Exo
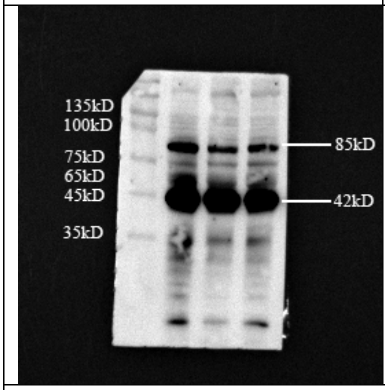	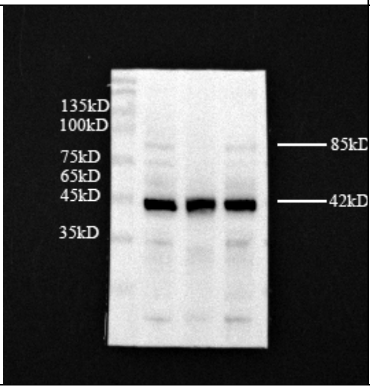	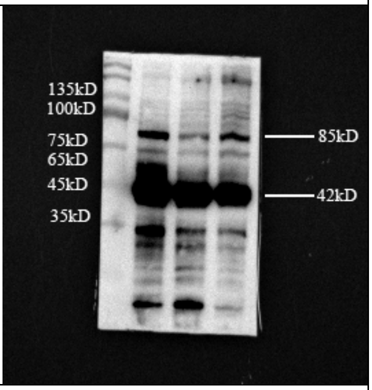
actin-pig-1d		
Repeat 1	Repeat 2	Repeat 3
Sham IRI Exo	Sham IRI Exo	Sham IRI Exo
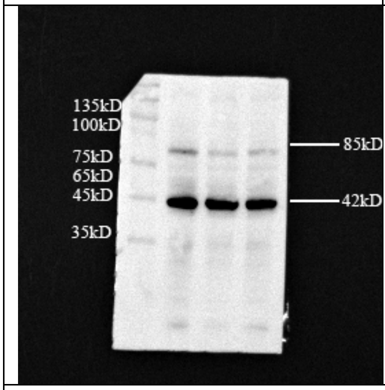	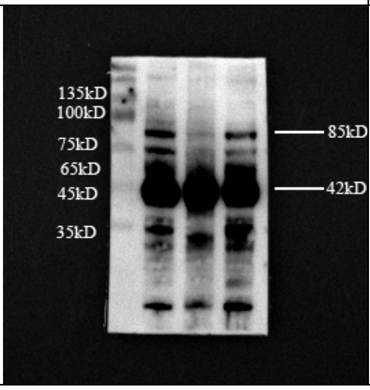	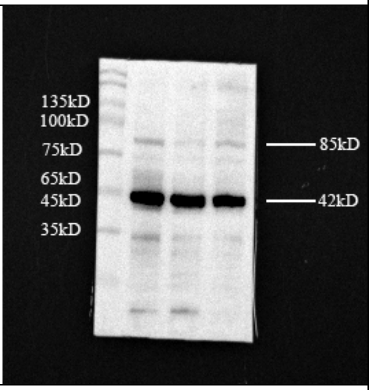
ACO2-pig-3d		
Repeat 1	Repeat 1	Repeat 1
Sham IRI Exo	Sham IRI Exo	Sham IRI Exo
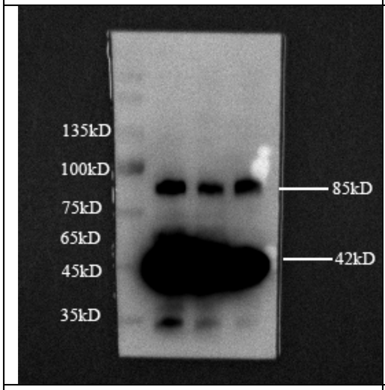	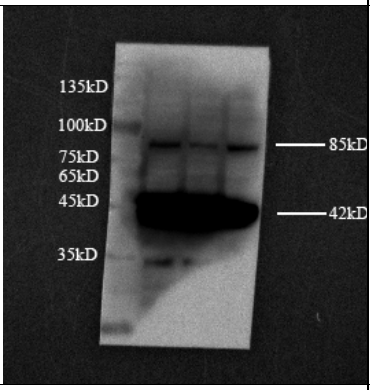	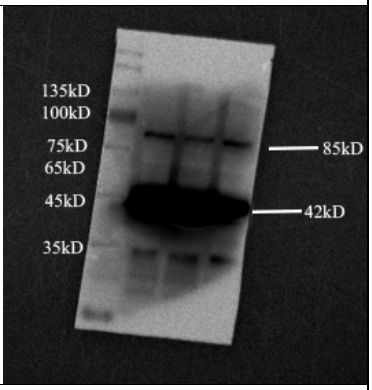

actin-pig-3d		
Repeat 1	Repeat 1	Repeat 1
Sham IRI Exo	Sham IRI Exo	Sham IRI Exo

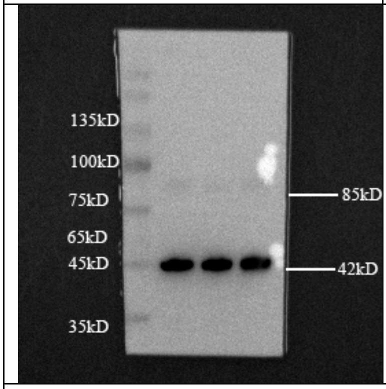

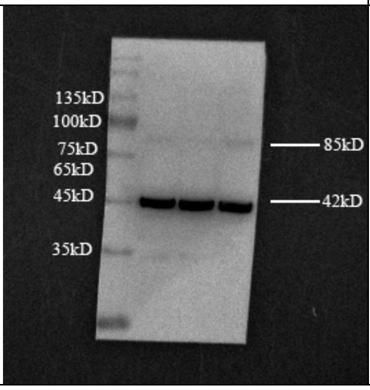

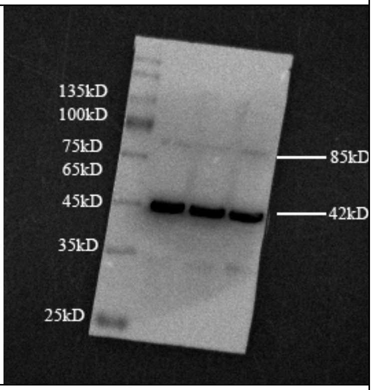

ACO2-pig-7d		
Repeat 1	Repeat 1	Repeat 1
Sham IRI Exo	Sham IRI Exo	Sham IRI Exo

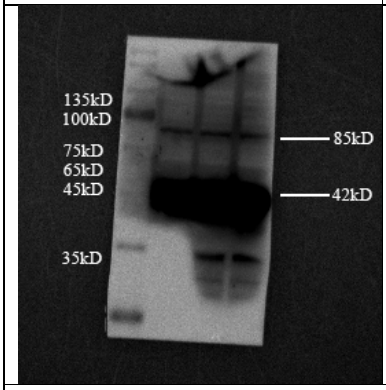

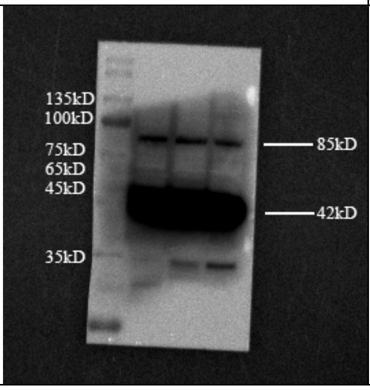

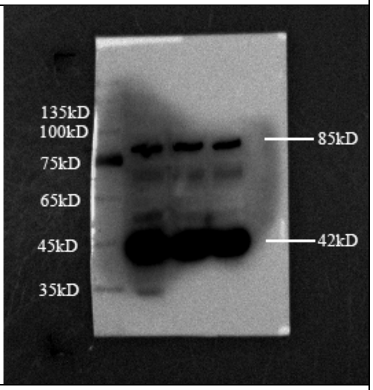

actin-pig-7d		
Repeat 1	Repeat 1	Repeat 1
Sham IRI Exo	Sham IRI Exo	Sham IRI Exo

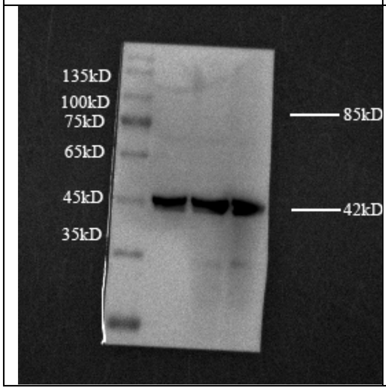

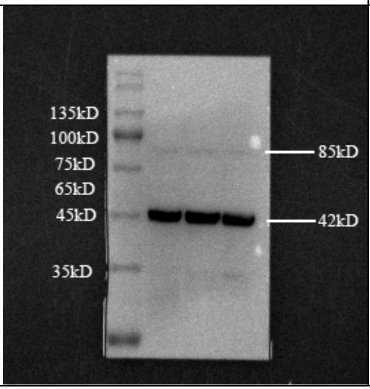

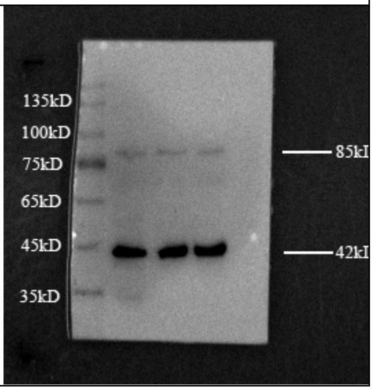

Original image of SDHB expression in pig tissues detected by Western blot

SDHB-pig-1d		
Repeat 1	Repeat 2	Repeat 3
Sham IRI Exo	Sham IRI Exo	Sham IRI Exo

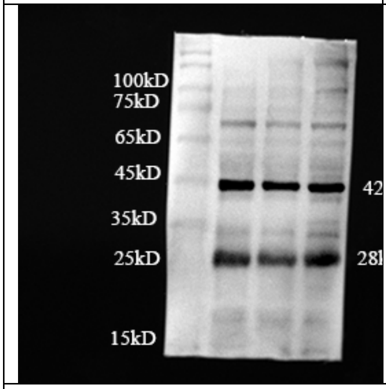

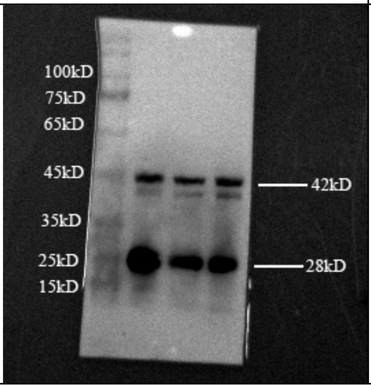

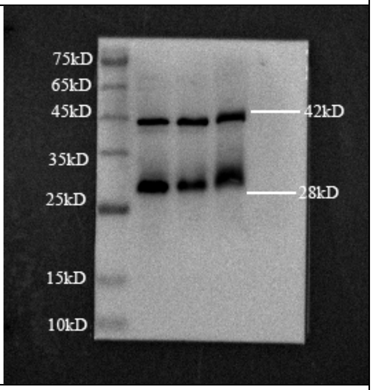

actin-pig-1d		
Repeat 1	Repeat 2	Repeat 3
Sham IRI Exo	Sham IRI Exo	Sham IRI Exo

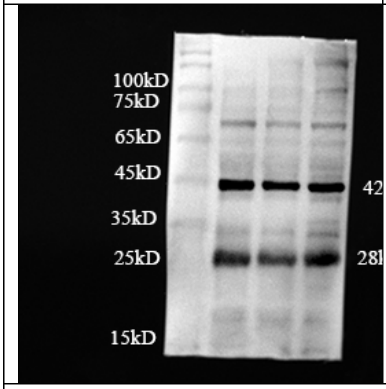

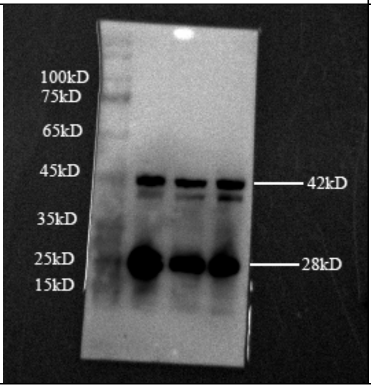

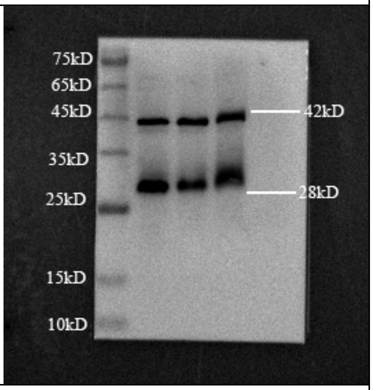

SDHB-pig-3d		
Repeat 1	Repeat 1	Repeat 1
Sham IRI Exo	Sham IRI Exo	Sham IRI Exo

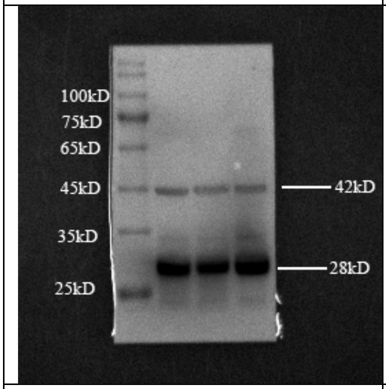

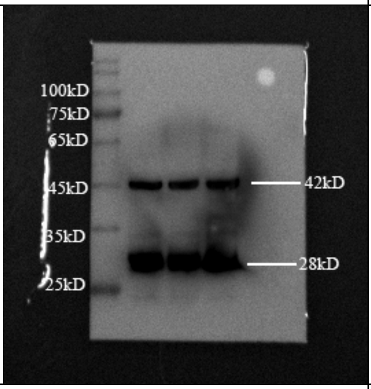

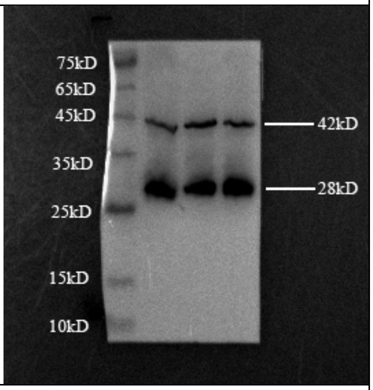

actin-pig-3d		
Repeat 1	Repeat 1	Repeat 1
Sham IRI Exo	Sham IRI Exo	Sham IRI Exo
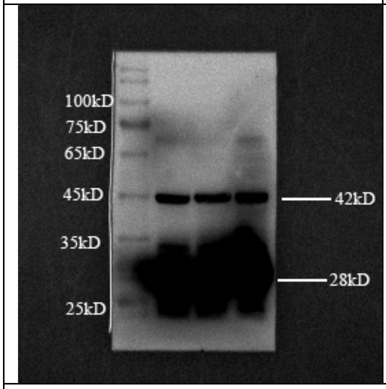	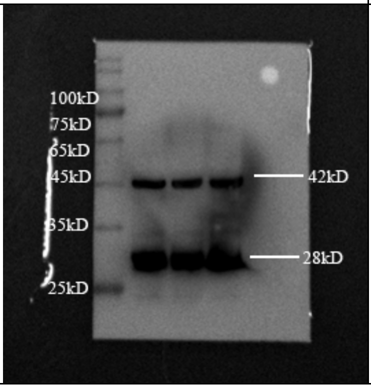	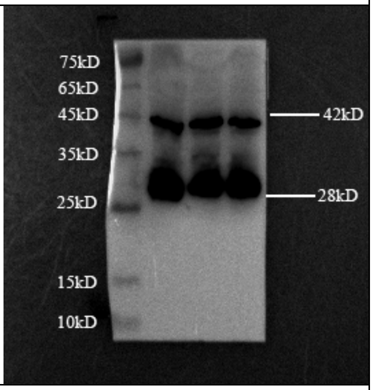
SDHB-pig-7d		
Repeat 1	Repeat 1	Repeat 1
Sham IRI Exo	Sham IRI Exo	Sham IRI Exo
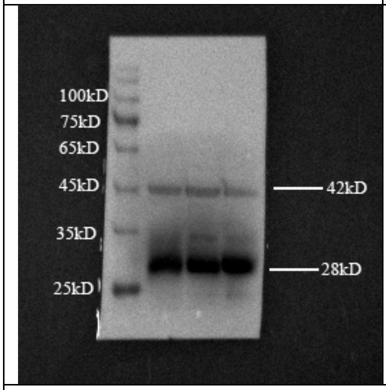	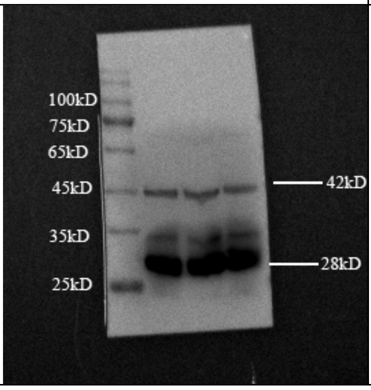	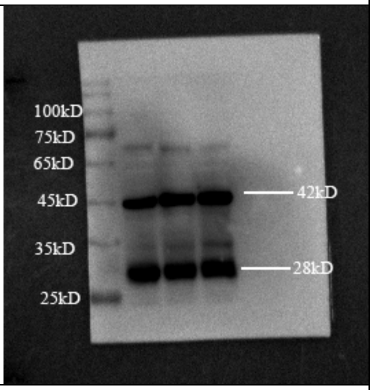
actin-pig-7d		
Repeat 1	Repeat 1	Repeat 1
Sham IRI Exo	Sham IRI Exo	Sham IRI Exo
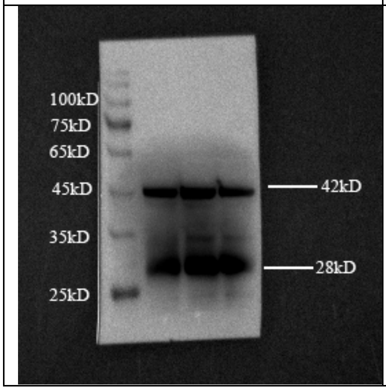	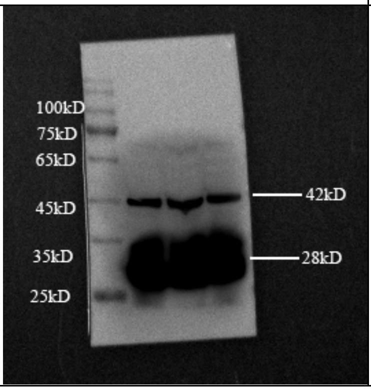	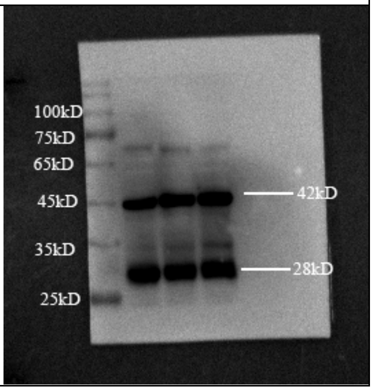

Original image of HSP70 expression in pig tissues detected by Western blot

<table>
<tr><td colspan="3">HSP70-pig-1d</td></tr>
<tr><td>Repeat 1</td><td>Repeat 2</td><td>Repeat 3</td></tr>
<tr><td>Sham IRI Exo</td><td>Sham IRI Exo</td><td>Sham IRI Exo</td></tr>
<tr><td>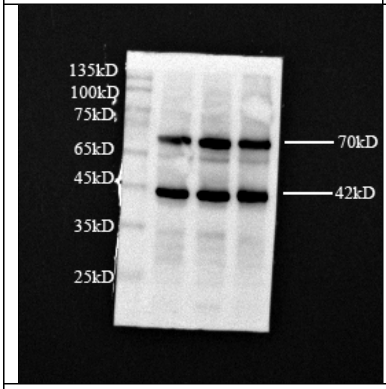
</td><td>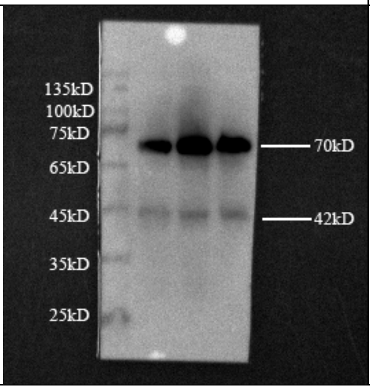
</td><td>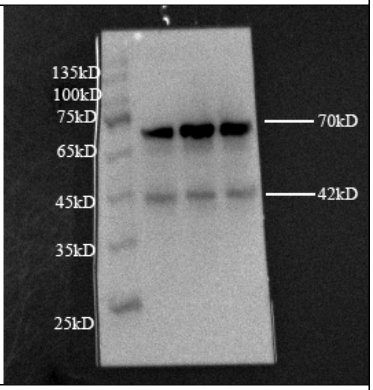
</td></tr>
<tr><td colspan="3">actin-pig-1d</td></tr>
<tr><td>Repeat 1</td><td>Repeat 2</td><td>Repeat 3</td></tr>
<tr><td>Sham IRI Exo</td><td>Sham IRI Exo</td><td>Sham IRI Exo</td></tr>
<tr><td>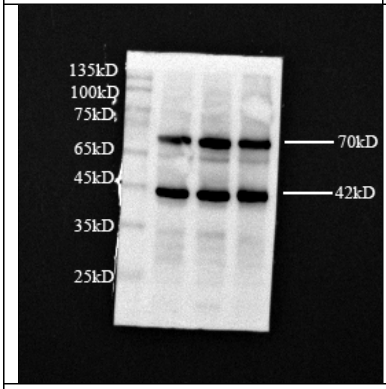
</td><td>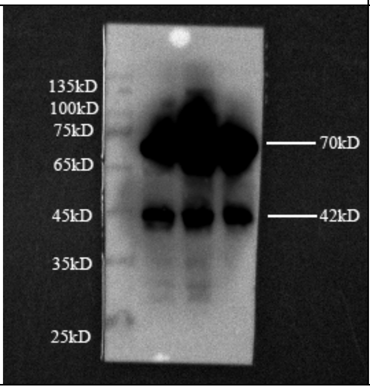
</td><td>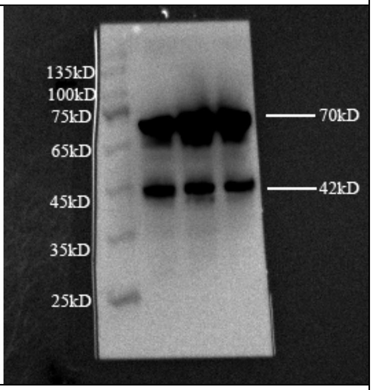
</td></tr>
<tr><td colspan="3">HSP70-pig-3d</td></tr>
<tr><td>Repeat 1</td><td>Repeat 1</td><td>Repeat 1</td></tr>
<tr><td>Sham IRI Exo</td><td>Sham IRI Exo</td><td>Sham IRI Exo</td></tr>
<tr><td>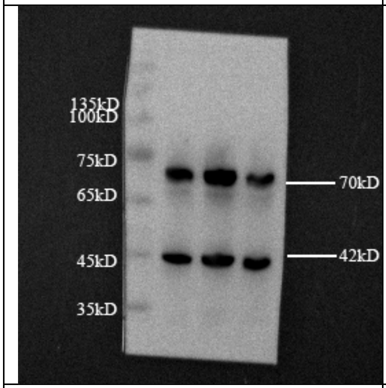
</td><td>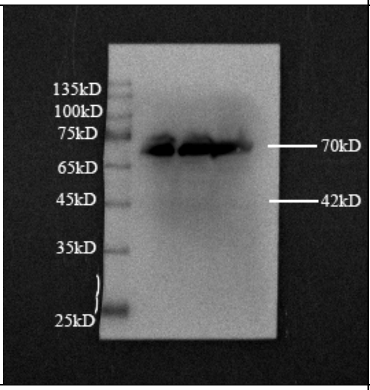
</td><td>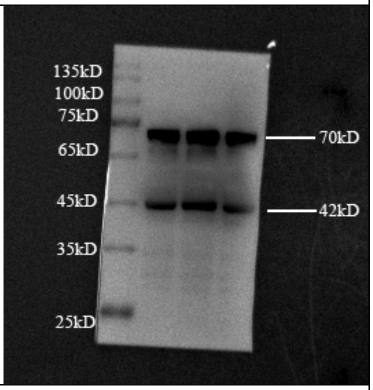
</td></tr>
<tr><td></td><td></td><td></td></tr>
</table>

<table>
<tr><td colspan="3">actin-pig-3d</td></tr>
<tr><td>Repeat 1</td><td>Repeat 1</td><td>Repeat 1</td></tr>
<tr><td>Sham IRI Exo</td><td>Sham IRI Exo</td><td>Sham IRI Exo</td></tr>
<tr><td>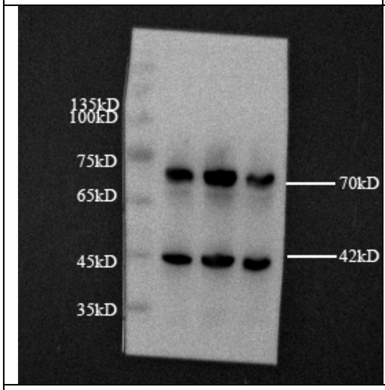
</td><td>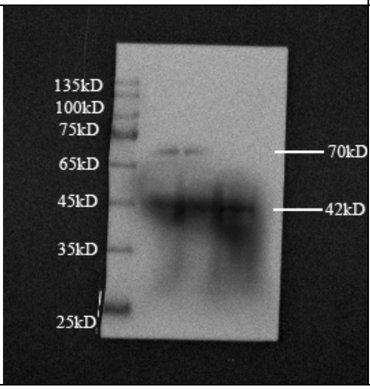
</td><td>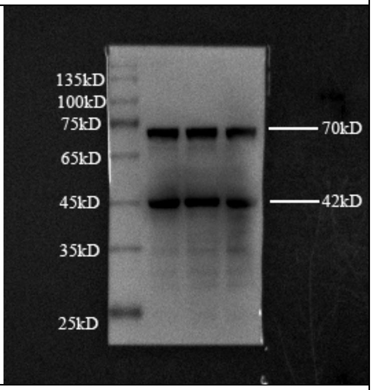
</td></tr>
<tr><td colspan="3">HSP70-pig-7d</td></tr>
<tr><td>Repeat 1</td><td>Repeat 1</td><td>Repeat 1</td></tr>
<tr><td>Sham IRI Exo</td><td>Sham IRI Exo</td><td>Sham IRI Exo</td></tr>
<tr><td>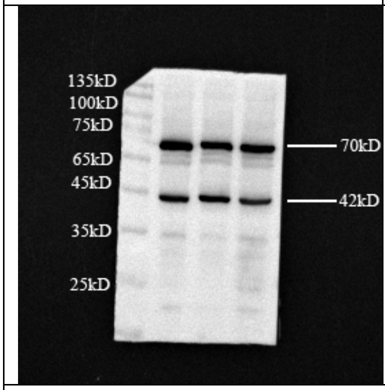
</td><td>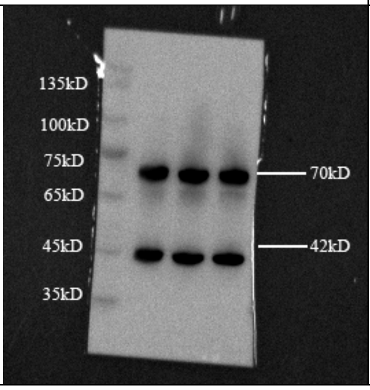
</td><td>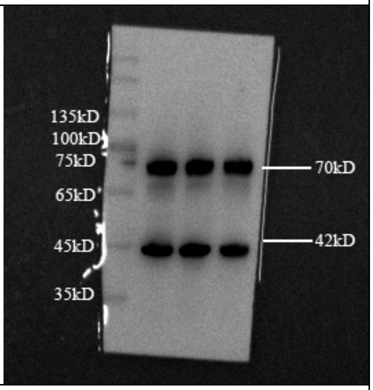
</td></tr>
<tr><td colspan="3">actin-pig-7d</td></tr>
<tr><td>Repeat 1</td><td>Repeat 1</td><td>Repeat 1</td></tr>
<tr><td>Sham IRI Exo</td><td>Sham IRI Exo</td><td>Sham IRI Exo</td></tr>
<tr><td>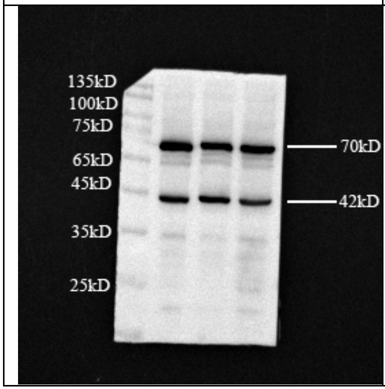
</td><td>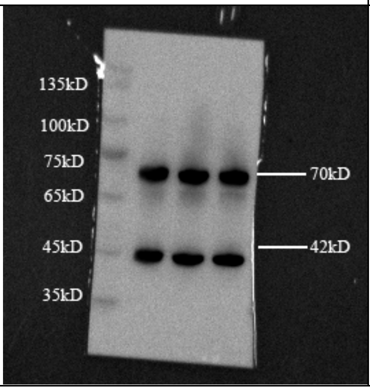
</td><td>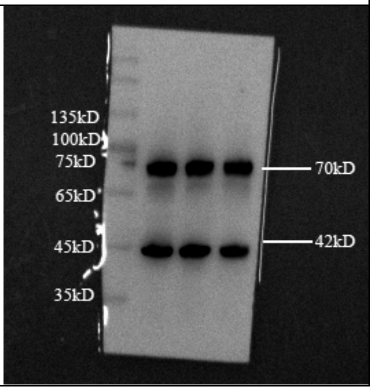
</td></tr>
</table>

Original image of LIP-DLAT expression in pig tissues detected by Western blot

LIP-DLAT-pig-1d

Repeat 1	Repeat 2	Repeat 3
Sham IRI Exo	Sham IRI Exo	Sham IRI Exo

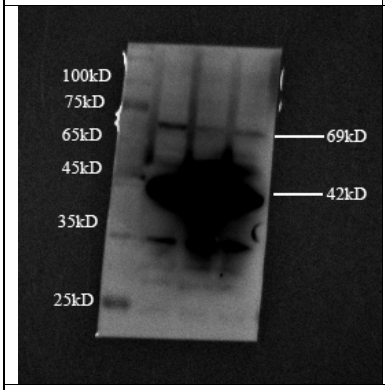

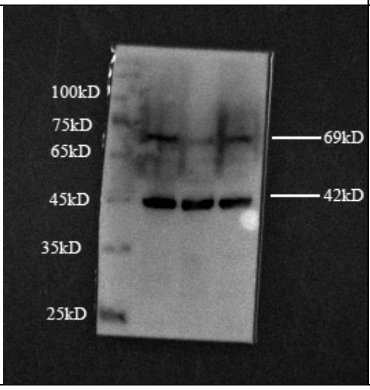

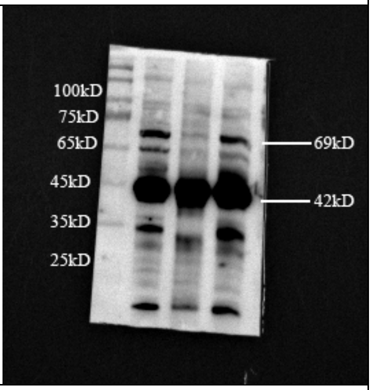

actin-pig-1d

Repeat 1	Repeat 2	Repeat 3
Sham IRI Exo	Sham IRI Exo	Sham IRI Exo

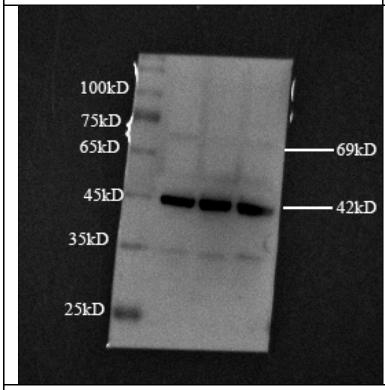

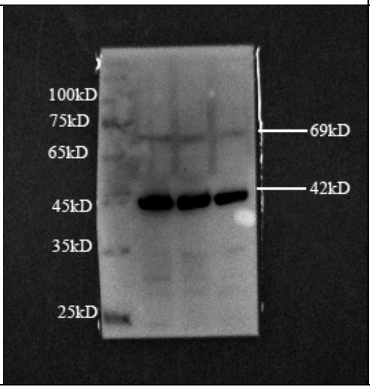

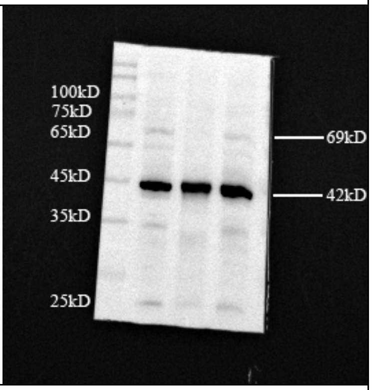

LIP-DLAT-pig-3d

Repeat 1	Repeat 1	Repeat 1
Sham IRI Exo	Sham IRI Exo	Sham IRI Exo

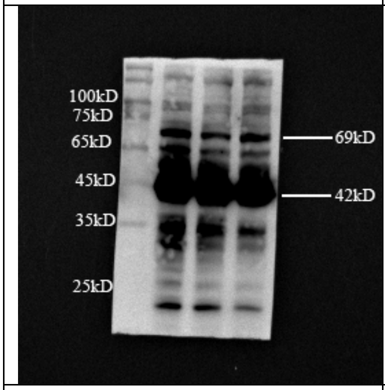

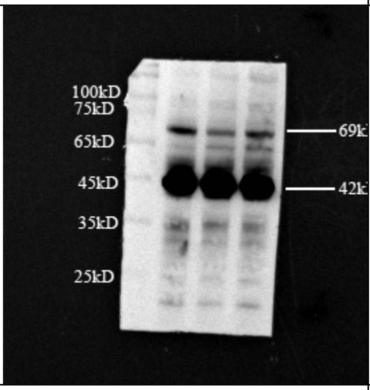

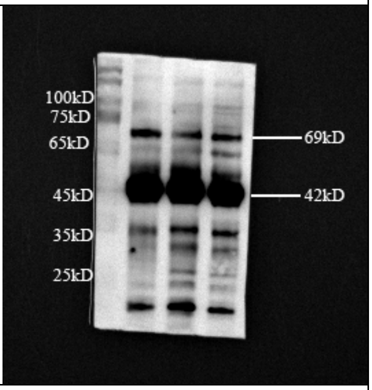

actin-pig-3d		
Repeat 1	Repeat 1	Repeat 1
Sham IRI Exo	Sham IRI Exo	Sham IRI Exo

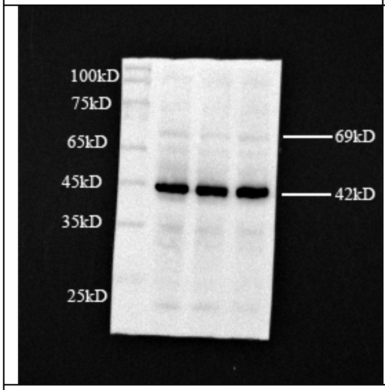

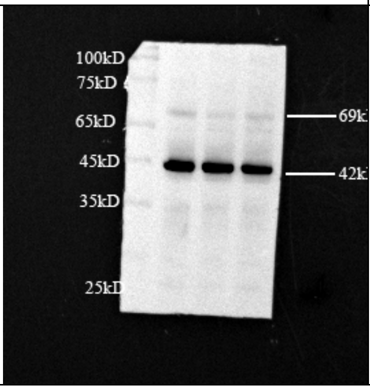

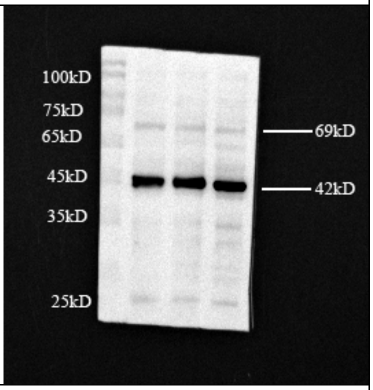

LIP-DLAT-pig-7d		
Repeat 1	Repeat 1	Repeat 1
Sham IRI Exo	Sham IRI Exo	Sham IRI Exo

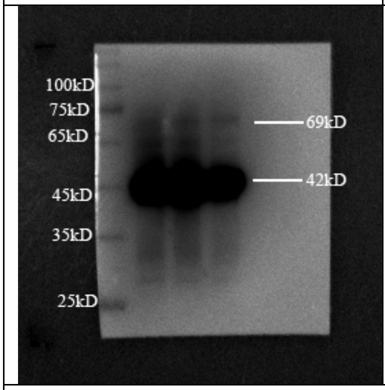

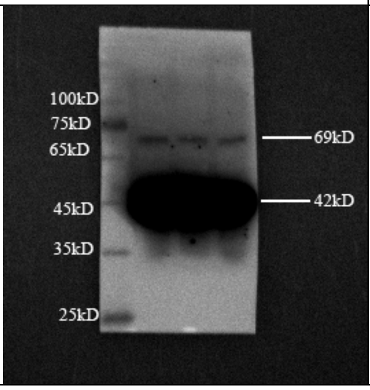

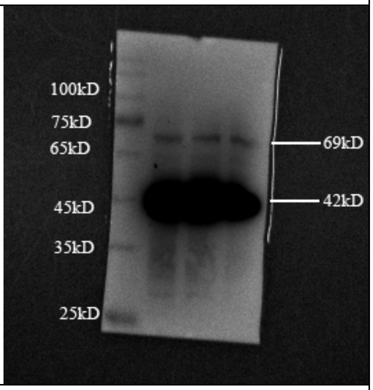

actin-pig-7d		
Repeat 1	Repeat 1	Repeat 1
Sham IRI Exo	Sham IRI Exo	Sham IRI Exo

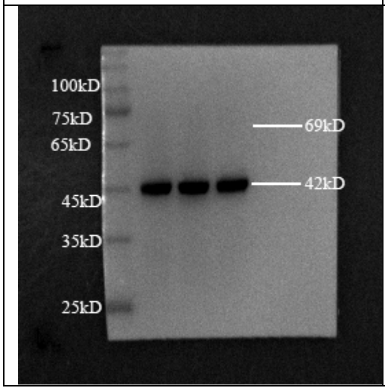

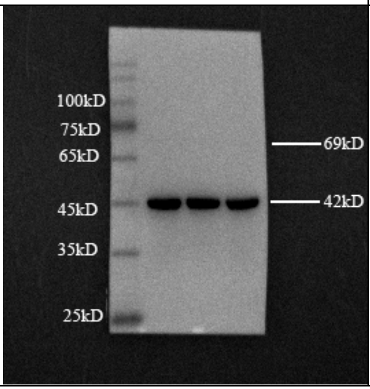

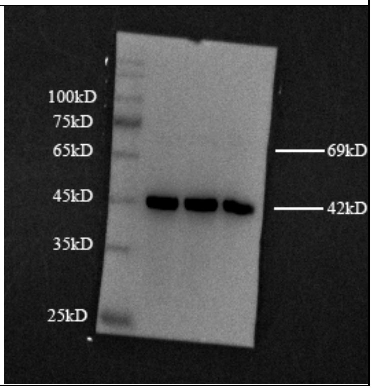

Original image of DLAT expression in pig tissues detected by Western blot

<table>
<tr><td colspan="3">DLAT-pig-1d</td></tr>
<tr><td>Repeat 1</td><td>Repeat 2</td><td>Repeat 3</td></tr>
<tr><td>Sham IRI Exo</td><td>Sham IRI Exo</td><td>Sham IRI Exo</td></tr>
<tr>
<td>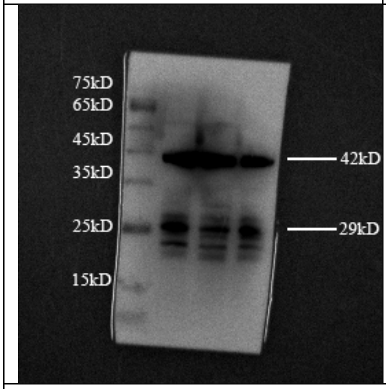
</td>
<td>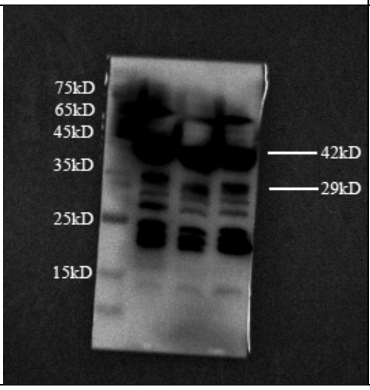
</td>
<td>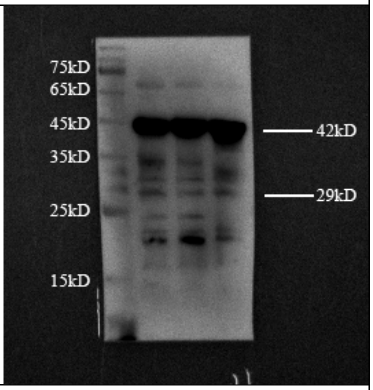
</td>
</tr>
<tr><td colspan="3">actin-pig-1d</td></tr>
<tr><td>Repeat 1</td><td>Repeat 2</td><td>Repeat 3</td></tr>
<tr><td>Sham IRI Exo</td><td>Sham IRI Exo</td><td>Sham IRI Exo</td></tr>
<tr>
<td>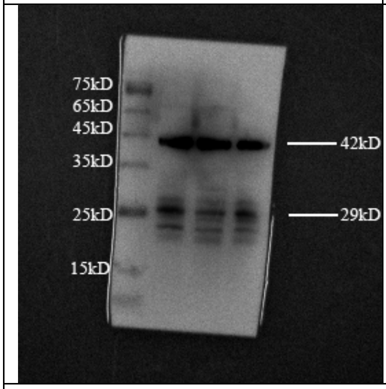
</td>
<td>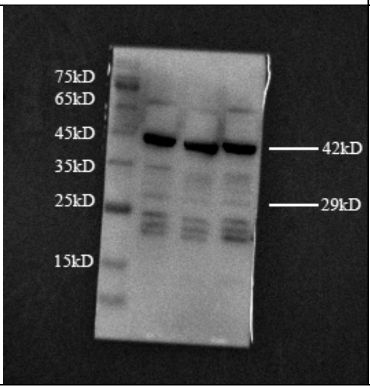
</td>
<td>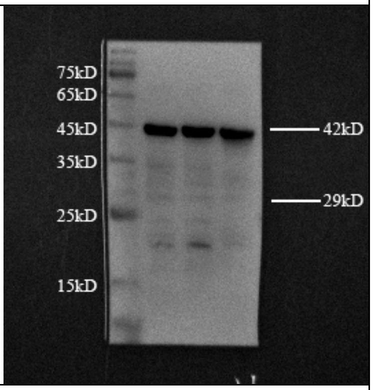
</td>
</tr>
<tr><td colspan="3">DLAT-pig-3d</td></tr>
<tr><td>Repeat 1</td><td>Repeat 1</td><td>Repeat 1</td></tr>
<tr><td>Sham IRI Exo</td><td>Sham IRI Exo</td><td>Sham IRI Exo</td></tr>
<tr>
<td>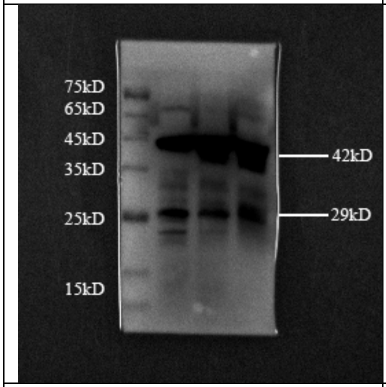
</td>
<td>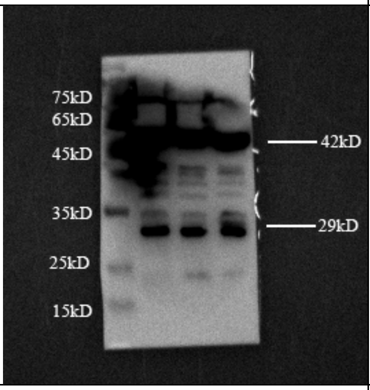
</td>
<td>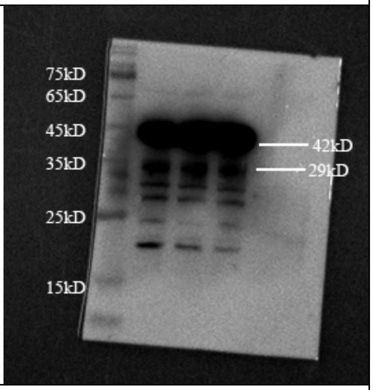
</td>
</tr>
<tr><td></td><td></td><td></td></tr>
</table>

actin-pig-3d		
Repeat 1	Repeat 1	Repeat 1
Sham IRI Exo	Sham IRI Exo	Sham IRI Exo

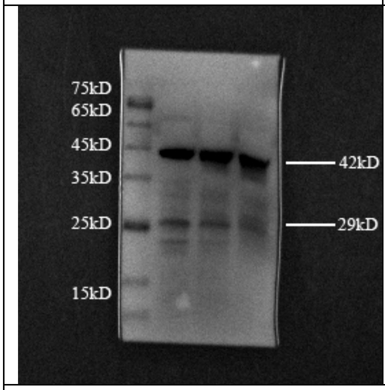

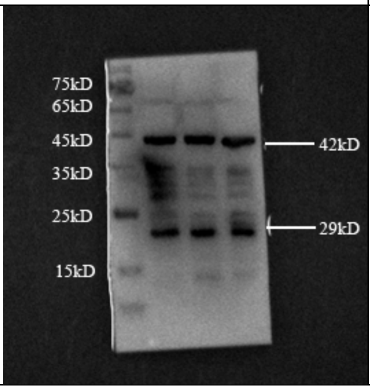

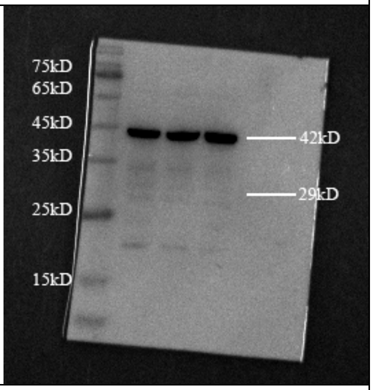

DLAT-pig-7d		
Repeat 1	Repeat 1	Repeat 1
Sham IRI Exo	Sham IRI Exo	Sham IRI Exo

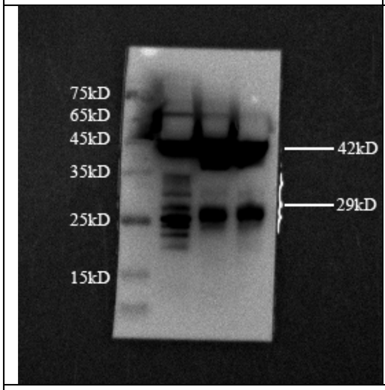

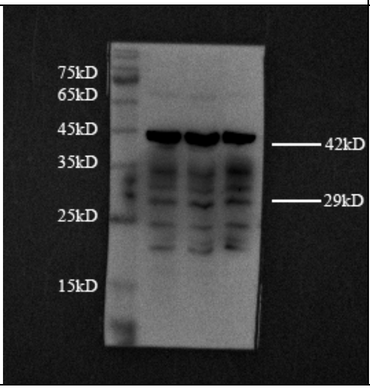

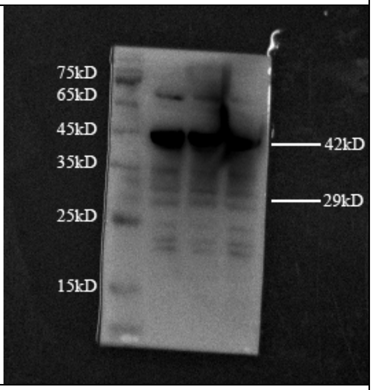

actin-pig-7d		
Repeat 1	Repeat 1	Repeat 1
Sham IRI Exo	Sham IRI Exo	Sham IRI Exo

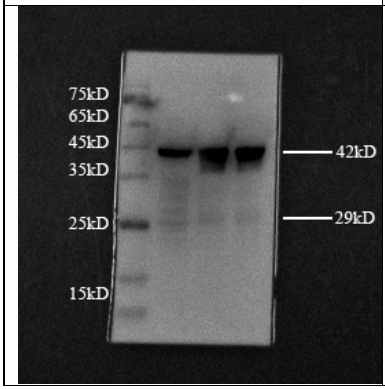

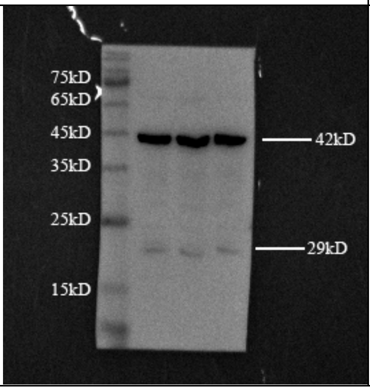

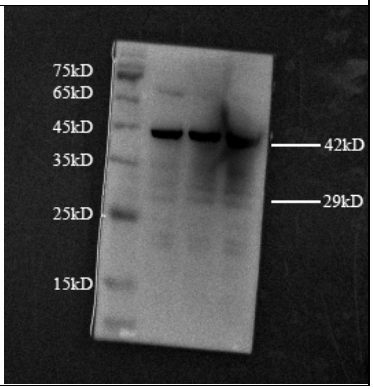

Original image of DLST expression in pig tissues detected by Western blot

<table>
<tr><td colspan="3">DLST-pig-1d</td></tr>
<tr><td>Repeat 1</td><td>Repeat 2</td><td>Repeat 3</td></tr>
<tr><td>Sham IRI Exo</td><td>Sham IRI Exo</td><td>Sham IRI Exo</td></tr>
<tr><td>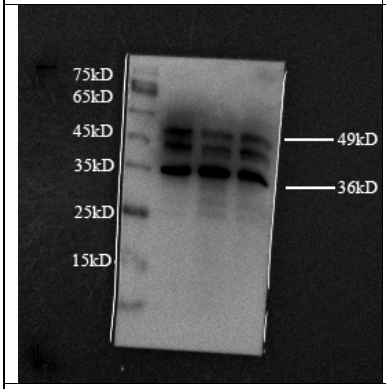</td><td>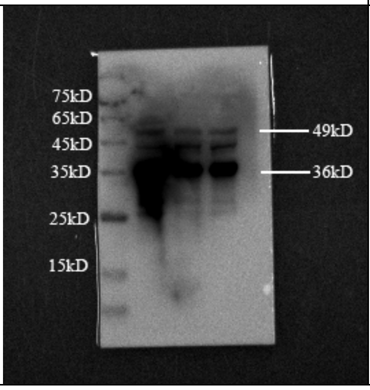</td><td>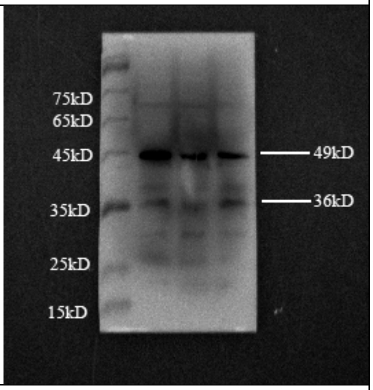</td></tr>
<tr><td colspan="3">GAPDH-pig-1d</td></tr>
<tr><td>Repeat 1</td><td>Repeat 2</td><td>Repeat 3</td></tr>
<tr><td>Sham IRI Exo</td><td>Sham IRI Exo</td><td>Sham IRI Exo</td></tr>
<tr><td>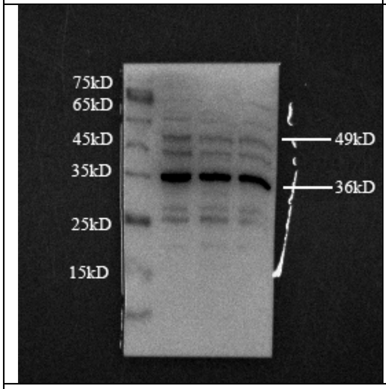</td><td>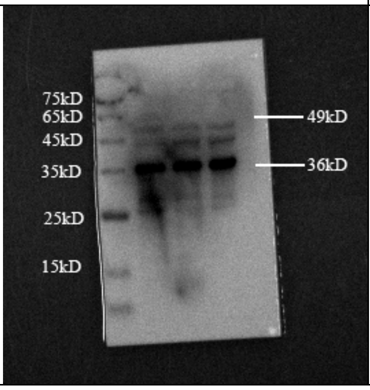</td><td>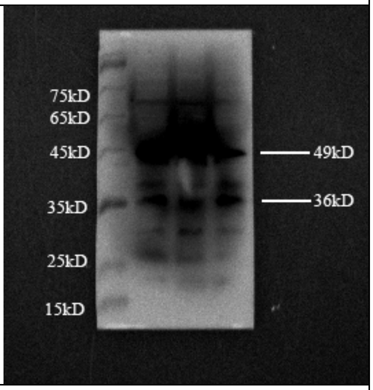</td></tr>
<tr><td colspan="3">DLST-pig-3d</td></tr>
<tr><td>Repeat 1</td><td>Repeat 1</td><td>Repeat 1</td></tr>
<tr><td>Sham IRI Exo</td><td>Sham IRI Exo</td><td>Sham IRI Exo</td></tr>
<tr><td>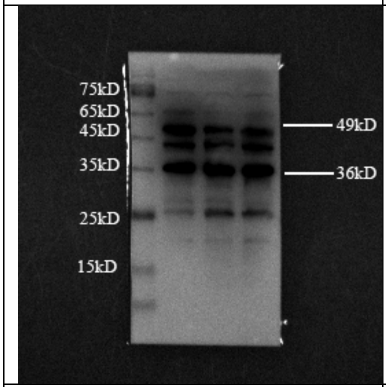</td><td>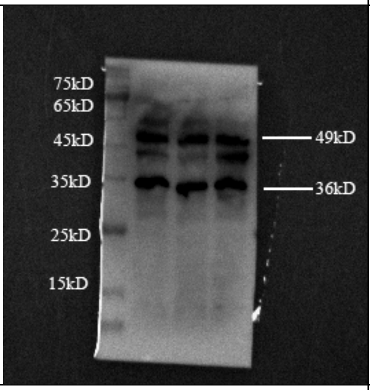</td><td>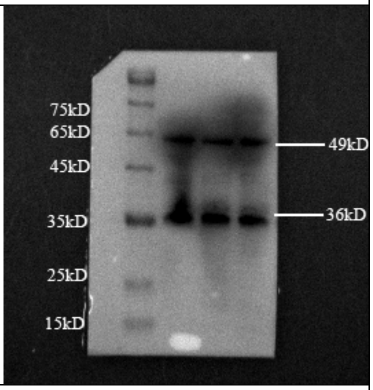</td></tr>
<tr><td></td><td></td><td></td></tr>
</table>

GAPDH-pig-3d

Repeat 1	Repeat 1	Repeat 1
Sham IRI Exo	Sham IRI Exo	Sham IRI Exo

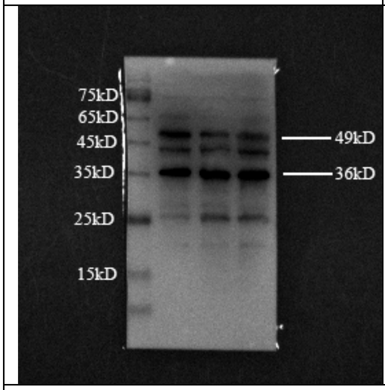

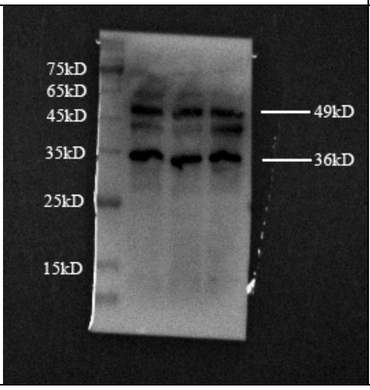

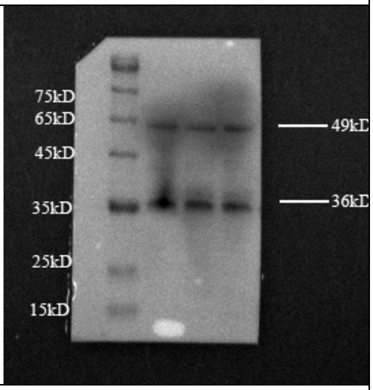

DLST-pig-7d

Repeat 1	Repeat 1	Repeat 1
Sham IRI Exo	Sham IRI Exo	Sham IRI Exo

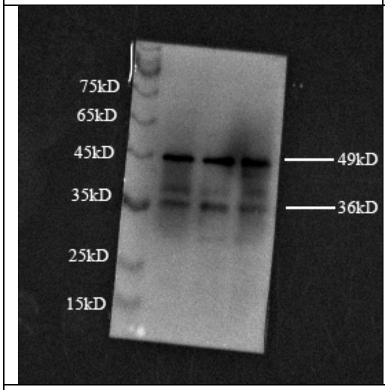

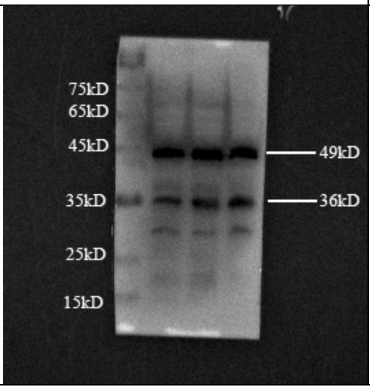

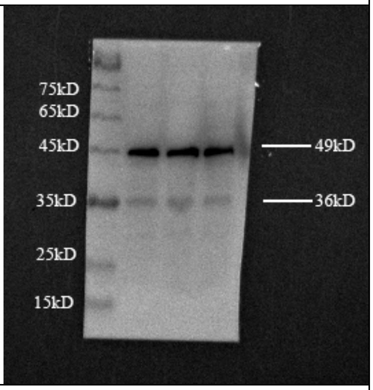

GAPDH-pig-7d

Repeat 1	Repeat 1	Repeat 1
Sham IRI Exo	Sham IRI Exo	Sham IRI Exo

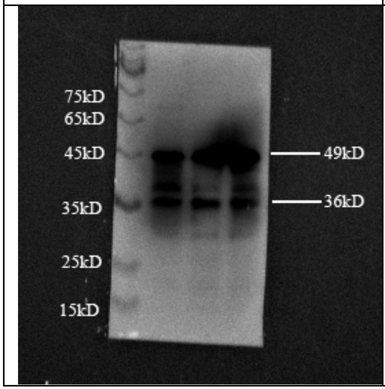

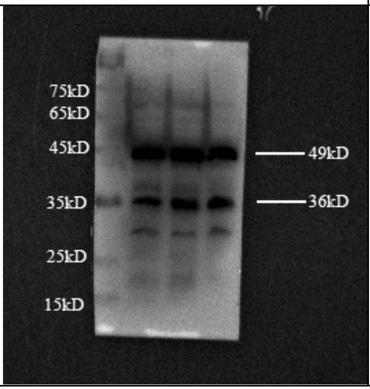

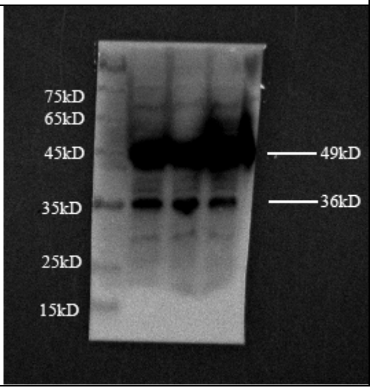

Supplement: Supplementary file 2 [file Data_sheet_2.pdf]
