# Supplementary material for: Adipose-derived mesenchymal stem cell exosomes ameliorate copper metabolism dysregulation and reduce cuproptosis caused by liver IRI
Source: Front Vet Sci. 2026 Jul 15;13:1895340. doi: 10.3389/fvets.2026.1895340 (PMC13414201; doi:10.3389/fvets.2026.1895340)
Supplement: Supplementary file 3 [file Table_1.docx]

**Supplemental Figure 1**


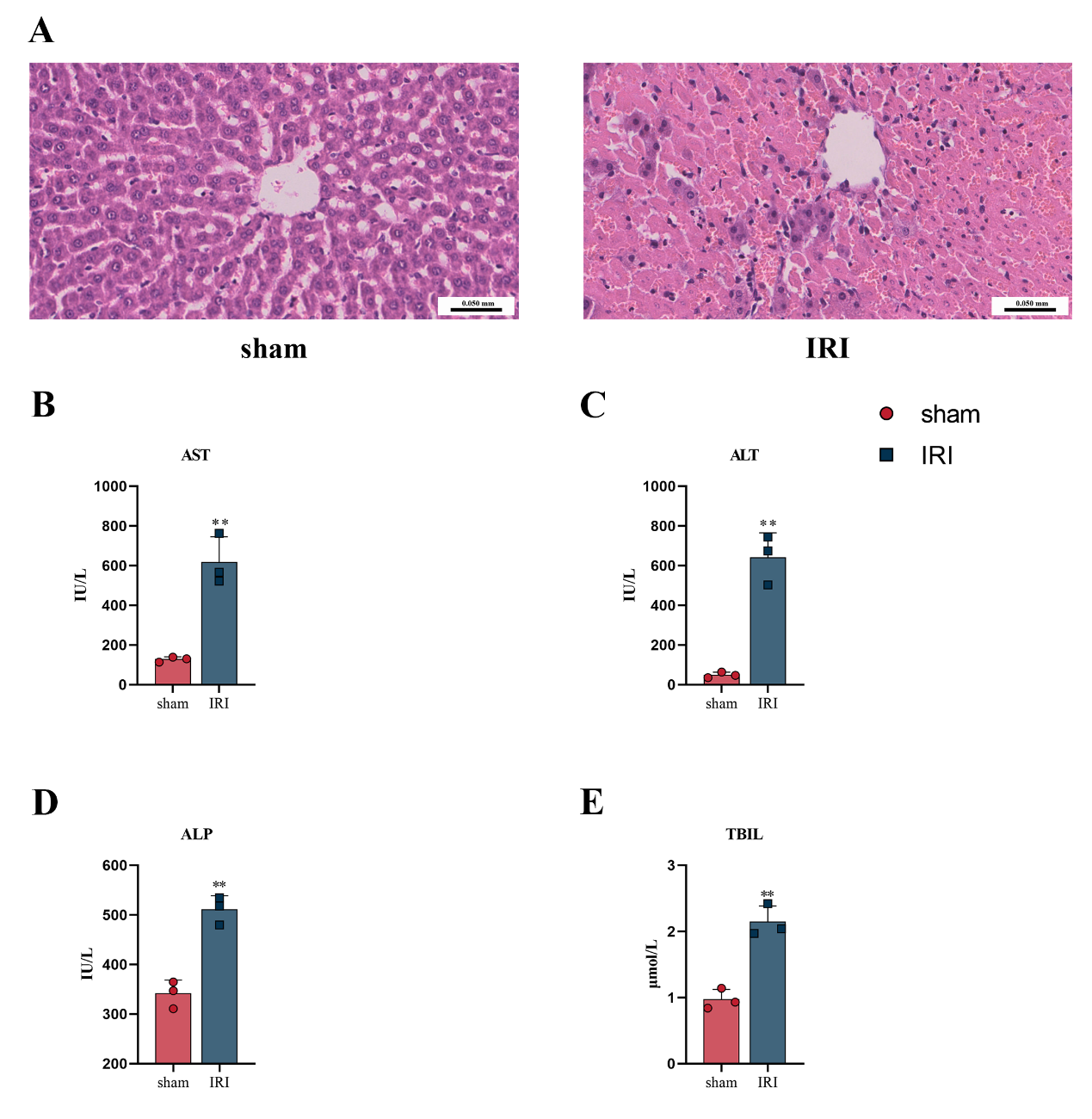


Supplementary Figure 1. Liver IRI damages the overall structure of liver tissue in rats and affects the normal function of liver. (A) Representative H&E staining (original magnification, ×400). (B-E) AST, ALT, ALP and TBIL level in the serum. Results are presented as mean ± SD (n=3) * *p*< 0.05, * * *p* < 0.01 versus the Sham group, ^#^ *p* < 0.05, ^##^ *p* < 0.01 versus the IRI group.

**Supplemental Figure 2**


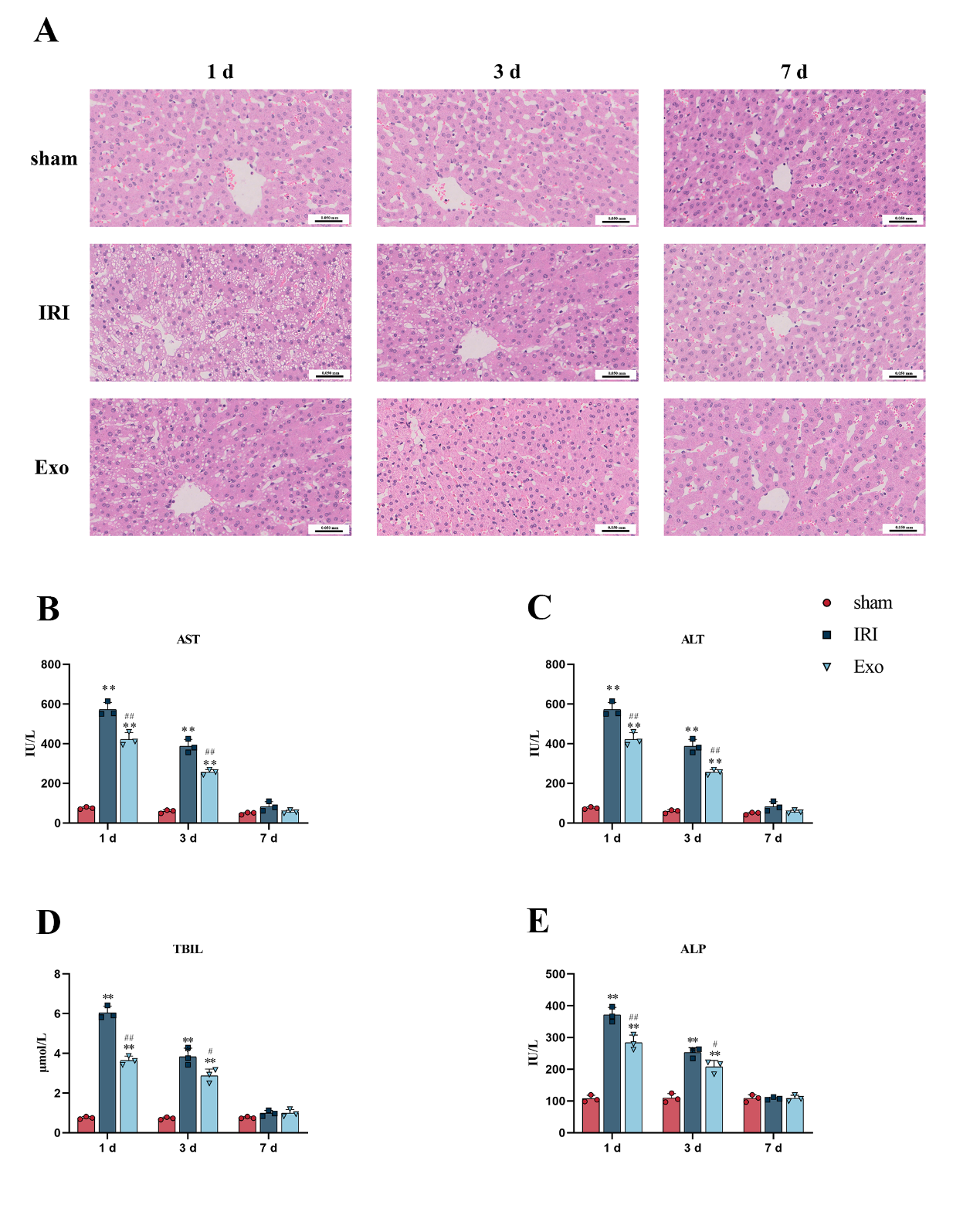


Supplementary Figure 2. ADSC-Exos effectively reduced the overall liver IRI injury and promoted the recovery of liver function in miniature pigs. (A) Representative H&E staining (original magnification, ×400). (B-E) AST, ALT, ALP and TBIL level in the serum. Results are presented as mean ± SD (n=3) * p< 0.05, * * p < 0.01 versus the Sham group, # p < 0.05, ## p < 0.01 versus the IRI group.

# **Supplementary Methods**

Supplementary Tables 1

Rat gene-specific primers were used for RT-qPCR.

| Gene | Forward primer sequence (5′-3′) | Reverse primer sequence (5′-3′) |
| --- | --- | --- |
| FDX1 | GAACCGAGATGGTGAAACGC | CCTCACACGCACCAAATCCA |
| LIAS | ATCCTCGTGGAATGCCTCAC | GGGATCACGGACTTTCCTCTG |
| ACO2 | GACTCCATCTCCTGCACTGG | AGGTTGGCAATGTCTGCTCG |
| SDHB | GCAGGCTTATCGCTGGATGA | CTGGATTCAGACCCTTGGGG |
| DLAT | GCCTACTAAGGCTGCTCCTG | CCTCTGCGCAATCACTCGAC |
| DLST | TCCAGGGTCTGTGGTCCTTT | TTCCACCAGGACAGTCAACG |
| CTR1 | CTCATCATCACCCAACCTCTTC | ATCACCAAACCAGCAAACAATAC |
| ATP7B | ATGGTGTGCTCTGTGGGATG | TCTTGTGGTTGTCTCCCGTG |
| HSP70 | GGTCTCAAGGGCAAGATCAG | TTTCTCAGCCAGCGTGTTAG |
| ATOX1 | CACGAGTTCTCTGTGGACAT | CAATGCAGACCTTCTTGTTGG |
| COX17 | TCAGGAGAAGAAGCCTCTGA | CGATGAGATGTCCACAGTGTT |
| CCS | TGGTTGTTGATGAGGGAGAAG | AGCAGAGCGTGCAATGAT |
| β-actin | TGTCACCAACTGGGACGATA | GGGGTGTTGAAGGTCTCAAA |

Supplementary Tables 2

Pig gene-specific primers were used for RT-qPCR.

| Gene | Forward primer sequence (5′-3′) | Reverse primer sequence (5′-3′) |
| --- | --- | --- |
| FDX1 | TGGCTTGCTCTACCTGTCAC | CCCAGCCGTGATCTATCTGT |
| LIAS | TGTATGCCCCCGGGTATTTG | TCATCCCAGGTGCTCTTGTC |
| ACO2 | ACAGCTGTGTGCAAGGATGAT | ATCACGCCATTTGCTGGTGA |
| SDHB | CCGCATCAGAAGGTTCCATT | TAACACTCCTCCAGGCTCTCA |
| DLAT | GATCGGCACTGATTCCCACA | TCACACCAATCACCTTGGGG |
| DLST | GAGCGCGAGAAACTGGATGG | GGAGTCGATCATCCAGCGAT |
| CTR1 | CACACAAAACAGTTGGGCAG | CACAGGTACCCGTTGTAGGT |
| ATP7B | ACTGGCTGACCGGTTTAGTG | CGCTTGCTGTGGTTAGGAAAG |
| HSP70 | GACGTGTCGGTCCTTACTATTG | ACCAAACGGTTGTCGAAGT |
| ATOX1 | GGTCCTCAACAAGCTGGGAG | ACGCTGTGCTCAGAGTCAAT |
| COX17 | CTGCCCGGAGACCAAGAAAG | TGTGGGCCTCAATTAGGTGTC |
| CCS | GGGTAGTGCGCTTCCTACAG | CCGTGAGACATCCCATCAGG |
| β-actin | TCTGGCACCACACCTTCT | TGATCTGGGTCATCTTCTCAC |
